# Targeting the NF-κB pathway enhances responsiveness of mammary tumors to JAK inhibitors

**DOI:** 10.1038/s41598-023-32321-0

**Published:** 2023-04-01

**Authors:** Aditi S. Bapat, Christine H. O’Connor, Kathryn L. Schwertfeger

**Affiliations:** 1 Molecular Pharmacology and Therapeutics Graduate Program, University of Minnesota, 2231 6th St SE, Minneapolis, MN 55455 USA; 2 University of Minnesota Supercomputing Institute, University of Minnesota, Minneapolis, MN USA; 3 Department of Laboratory Medicine and Pathology, University of Minnesota, Minneapolis, MN USA; 4 Masonic Cancer Center, University of Minnesota, Minneapolis, MN USA; 5 Center for Immunology, University of Minnesota, Minneapolis, MN USA

**Keywords:** Cancer, Immunology, Molecular biology, Oncology

## Abstract

Interactions between tumor cells and the tumor microenvironment are critical for tumor growth, progression, and response to therapy. Effective targeting of oncogenic signaling pathways in tumors requires an understanding of how these therapies impact both tumor cells and cells within the tumor microenvironment. One such pathway is the janus kinase (JAK)/signal transducer and activator or transcription (STAT) pathway, which is activated in both breast cancer cells and in tumor associated macrophages. This study demonstrates that exposure of macrophages to JAK inhibitors leads to activation of NF-κB signaling, which results in increased expression of genes known to be associated with therapeutic resistance. Furthermore, inhibition of the NF-κB pathway improves the ability of ruxolitinib to reduce mammary tumor growth in vivo. Thus, the impact of the tumor microenvironment is an important consideration in studying breast cancer and understanding such mechanisms of resistance is critical to development of effective targeted therapies.

## Introduction

Tumor associated macrophages (TAMs) are important components of the tumor microenvironment (TME) that greatly influence tumor growth and progression^1^. While the effects of macrophages on tumor cell behavior are complex and often context dependent, they are thought to correlate with poor prognosis in breast cancer^2,3^. TAMs regulate the growth and progression of the tumor either by directly influencing the tumor cells or by manipulating the TME by promoting angiogenesis, modifying the components of the extracellular matrix, promoting stem-ness, and by inhibiting immune response against the tumor^4^. Macrophages also mediate resistance of tumor cells to tumor-directed inhibitors, by activating compensatory survival pathways^5^. Several approaches for targeting macrophage recruitment, survival, and activation in the TME have been successful in pre-clinical models of cancer and have also led to investigations in clinical trials^5^.

In order to understand how macrophages contribute to therapeutic responsiveness in tumors, is it important to define how these inhibitors impact macrophage function. By virtue of their proximity to the tumor, inhibitors are likely to affect their target pathways in TAMs as well. The Janus Kinase (JAK)/Signal Transducer and Activator of Transcription (STAT) pathway is an oncogenic transcription factor pathway activated in tumor cells^6^. Due to extensive studies linking JAK/STAT signaling to oncogenic functions in cancer cells, JAK inhibitors are being investigated in the clinic for treatment of solid tumors and hematological malignancies^6^. The JAK proteins are associated with tyrosine kinase receptors activated by cytokines and growth factors^6^. Activated JAKs phosphorylate the STAT proteins, which in turn dimerize and translocate to the nucleus to activate several pathways involved in tumor cell survival, proliferation, and immune evasion^7^. More than 50% of tumor samples from breast cancer patients show evidence of activated JAK signaling^8^. The JAK/STAT pathway is of special interest in triple negative breast cancer since these tumors do not respond to conventional hormone-receptor targeted therapies^8^. Several inhibitors of JAK signaling, most prominently the JAK1/2 inhibitor ruxolitinib, are currently being investigated for solid tumors in clinical trials^9^. Ruxolitinib has been approved for treatment of rheumatoid arthritis and has shown promising results in clinical trials of myeloproliferative neoplasm, pancreatic cancer, and pre-clinical models of prostate cancer^10–14^. However, in pre-clinical models of breast cancer metastasis, ruxolitinib was shown to enhance metastasis due to decreased NK cell activation^15^. Ruxolitinib also inhibited T cell activation and differentiation in models of myeloproliferative neoplasms^16^. Given the promising therapeutic strategy presented by JAK inhibition, it is important to investigate its effect on the tumor stroma as a possible mechanism of resistance.

In addition to regulating tumor cells, the JAK/STAT pathway is also a critical regulator of macrophage function under normal and other pathophysiological conditions^17^. We have previously shown that tumor-cell derived factors from triple negative-like mammary tumor cell lines activate the JAK/STAT pathway in macrophages in culture and in mouse mammary tumor models^18^. Thus, JAK inhibitors are likely to have an effect on TAMs as well, consequently altering their behavior in the TME. Our studies with the JAK inhibitor ruxolitinib have shown that TAMs upregulate a subset of tumor-promoting genes in response to JAK inhibition by ruxolitinib. These genes are enriched in cancer-associated pathways and may be mediators of macrophage-derived resistance to ruxolitinib^18^. We sought to elucidate this phenomenon further and investigate the mechanism of macrophage mediated resistance to ruxolitinib. We demonstrate here that JAK inhibition in macrophages leads to activation of the Nuclear Factor kappa-B (NF-κB) transcription factor pathway.

One of the most prominent mediators of immune cell function, the NF-κB transcription factor pathway regulates transcription of several genes involved in the innate immune response^19^. Activation downstream of the tumor necrosis factor receptor (TNFR) is one of the most extensively studied mechanisms of NF-κB. However, several other stimuli are involved in the activation and regulation of the pathway^19^. The canonical activation pathway involves phosphorylation of the p65 (RelA) subunit of the NF-κB dimer, by upstream kinases including but not limited to inhibitor of kappa B (IκB) kinase IKKα/β, which targets the sequestering protein IκB for ubiquitination, and releases the p65-p50 dimer to translocate to the nucleus and regulate transcription^20^. In this study, we investigated NF-κB as a mechanism through which macrophages mediate resistance to JAK inhibition. Our studies show that ruxolitinib treatment in TAMs activates the NF-κB pathway, which upregulates transcription of a subset of genes. Combining an NF-κB inhibitor with ruxolitinib showed improved survival over either inhibitor alone. These findings highlight the importance of investigating the effects of targeted therapy on cells of the tumor stroma and targeting possible mechanisms of resistance.

## Materials and methods

### Mice

Wild-type (WT) Balb/c mice were obtained from Envigo. All experiments were performed with 8-week-old female mice and all animal care and procedures were approved by the Institutional Animal Care and Use Committee of the University of Minnesota and in accordance with the procedures detailed in the Guide for the Care and Use of Laboratory Animals^21^.

### Cell culture and stimulation

HC11/R1 cells were obtained from Jeffrey Rosen, Baylor College of Medicine, Houston, TX and maintained as described before^22^. 4T1 cells were obtained from Thomas Griffith, University of Minnesota, Minneapolis, MN and grown in media containing RPMI, 10% FBS, 1% penicillin/streptomycin (Life Technologies), 1% l-glutamine (Life Technologies), 10 mM HEPES (Life Technologies), 1 mM sodium pyruvate (Life Technologies). Mouse bone marrow-derived macrophages (BMDMs) were isolated and maintained as described previously^23^. Serum starved HC11/R1 cells were treated with 30 nM B/B homodimerizer (Clontech) for 24 h. 4T1 cells were serum starved for 24 h. Conditioned medium (CM) was collected from both cell lines and spun down to remove cell debris. Prior to CM treatment, BMDMs were serum starved for 2 h to downregulate any baseline activation of pathways. For BMDMs treated with CM and ruxolitinib, 0.5 μM ruxolitinib was added during pre-treatment to enable activation specifically by soluble factors from the CM. BMDMs were treated with conditioned media (CM) from HC11/R1 and 4T1 cells with ruxolitinib for 15 min for immunoblots, to observe rapid phosphorylation of proteins of interest, and 4 h for isolating RNA, to allow for transcriptional changes to occur after phosphorylation. Alternatively, BMDMs were treated with 5 μM JAK1 inhibitor solcitinib (MedKoo Biosciences) and 5 μM JAK2 inhibitor NVP-BSK085 (Tocris Biosciences) for 15 min with or without CM. In experiments with IKK-16, BMDMs were pre-treated as described above and 0.8 μM IKK-16 was added along with ruxolitinib and CM. For co-culture of macrophages with tumor cells to measure the number of surviving cells, tumor cells were plated in the bottom of a 24-well plate. BMDMs were plated in a 0.4 µm hanging insert. The tumor cells and macrophages were treated together with 0.5 µM ruxolitinib for 24 h. For treatments with different concentrations of ruxolitinib, tumor cells and macrophages were plated in coculture as described above and treated with 0.1, 0.5, 1.0, 5.0 and 10.0 µM for 24 h. For studying the effect of ruxolitinib independent of tumor-derived factors, macrophages were first treated with ruxolitinib for 4 h. Media was replaced after 4 h and macrophages were incubated for 24 more hours. This supernatant was then used to treat 4T1 and HC11/R1 cells in presence of ruxolitinib for 24 h. Cell survival for all inhibitor treatments was measured by staining the attached cells with Crystal Violet (0.5 mg/mL) and solubilized in 70% ethanol. Absorbance was measured at 590 nm.

### Nuclear fractionation

BMDMs were stimulated as described above. Nuclear and cytoplasmic proteins were separated 15 min after CM and ruxolitinib treatment using the NE-PER Nuclear and Cytoplasmic Extraction Kit (Thermo Fisher), according to manufacturer’s protocol. Protein lysates were analyzed by immunoblot as described below.

### Immunoblot analysis

Cells were lysed in RIPA buffer with added protease inhibitors and protein content was measured by BCA assay. Total protein was resolved using SDS-PAGE and subjected to immunoblot analysis. Nuclear and cytoplasmic protein fractions were separately processed similar to total protein and analyzed by immunoblot. Densitometric analyses were performed using ImageJ and phospho-p65 values were normalized to total p65. Uncropped blots are included with supplemental data. Antibodies used were phospho-STAT3 (1:1000, Cell Signaling #9131S), phospho-STAT5 (1:1000, Cell Signaling 9314S), phospho-p65 (1:1000, Cell Signaling #3033S), total STAT3 (1:1000, Cell Signaling #12640), total STAT5 (1:1000, Cell Signaling #94205), total p65 (1:1000, Cell Signaling #8242), GAPDH (1:10,000, Cell Signaling #2118), β-tubulin (1:1000, Cell Signaling #2146S), Lamin B1 (1:1000, Cell Signaling #12586S). We detected two bands for total STAT5 proteins, which correspond to Stat5a and STAT5b, isoforms of STAT5 encoded by homologous genes^24^. We also detected two bands for p65, which may correspond to one of the reported isoforms for p65, of which the larger one is transcriptionally active^25^.

### In vivo studies

For tumor studies, 10,000 4T1 cells in 50% Matrigel (BD Biosciences) were injected in mammary fat pads of 8-week-old WT Balb/c mice. Mice were examined for tumor growth by palpation. Once the tumor size reached 200 mm^3^ the mice were randomly assigned to one of 4 treatment groups: DMSO (control for ruxolitinib and IKK-16), Ruxolitinib (Astatech) (ruxolitinib and DMSO), IKK-16 (Astatech) (IKK-16 and DMSO) and Ruxolitinib + IKK-16. (n = 4 for ruxolitinib + DMSO treatment group, n = 5 for all others, total 19 WT mice were used for the experiment). Beginning treatment at 200 mm^3^ tumor size allows for establishment of the tumor, enables consistent accurate measurement of the tumors at the beginning of treatment, and also recapitulates clinical conditions where treatment is begun after patients present to the clinic with a detectable tumor. Tumor volumes were measured in a random sequence of cages each day, with treatment conditions being blinded. Ruxolitinib was resuspended in DMSO and diluted in 2% Tween20 in PBS for administration of 60 mg/kg as daily oral gavage. IKK-16 was resuspended in DMSO and diluted in 1% Tween20 in PBS for 30 mg/kg administered subcutaneously every alternate day^18,26,27^. Tumors were measured by calipers using the formula V = (L × W^2^)/2. Mice were euthanized when tumors reached 1000 mm^3^. Survival was plotted as number of days until tumor endpoint. Mice were included in the study if they underwent successful injection of tumor cells in the mammary fat pad. One experimental mouse did not get a successful injection of tumor cells and was excluded from drug treatments and further analysis. For tumor volume measurements and tissue processing for each animal, the investigator (AB) was blinded to the treatment group until after the completion of the analyses, with each mouse tracked on the basis of its random identifier. All experiments performed in the study were in accordance with ARRIVE guidelines^28^.

### Tissue analysis

All mice were injected with 30 mg/kg of 5-bromo-2’-deoxyuridine i.p 2 h prior to sacrifice. Tumors were fixed in 4% paraformaldehyde for 4 h and embedded in paraffin. 5 µm thick sections were stained with anti-BrdU antibody (1:200, Abcam) and DeadEnd Fluorometric TUNEL System (Promega).

### Microscope image acquisition

All Images were taken on a Keyence Fluorescence microscope BZ-X800E with a 20X objective. 5 images were randomly captured for each tumor section and number of BrdU and TUNEL positive cells per section were quantified using ImageJ.

### RNA-seq analysis

Total RNA was isolated using RNeasy Mini Kit (Qiagen) from mouse BMDMs treated with either 4T1 CM, CM with 0.5 μM ruxolitinib or CM with 0.5 μM ruxolitinib and 0.8 μM IKK-16. Samples were submitted in triplicate to the University of Minnesota Genomics Center. Library preparation was done through TruSeq Stranded mRNA Library Preparation and quality control was performed using Agilent Bioanalyzer/TapeStation analysis. Samples were multiplexed in one lane of NovaSeq S2 and 150 bp paired-ends were sequenced (20 M reads/sample). All samples passed quality control, containing > 500 ng of RNA and having an RIN above 8. Individual gene expression plots were obtained by calculating counts per million (CPM) for each gene. For downstream analysis, genes with LogFC > 0.5 and FDR < 0.05 were selected.

### RNA-seq data processing

Bulk RNA-seq samples were processed and aligned using the CHURP version 0.2.2 command line interface framework^29^. A full description of the CHURP pipeline can be found in Baller et al.^30^. Briefly, trimmomatic version 0.33 was used to clean reads for adapter contamination and low-quality sequence, and FastQC was used to generate sequence quality reports for raw and trimmed reads^31^. HISAT2 version 2.1.0 was used to align samples to the genome reference consortium mouse build 38 reference genome^32^. FeatureCounts v1.6.2 was used to count mapped reads to genes. *M. musculus* GRC build 38.100 gtf file was used^33^.

### Gene expression and pathway analysis

Differential gene expression analysis was done in EdgeR v 3.34.1^34^. Differentially expressed genes were identified between DMEM and CM treated samples, CM and CM + Ruxolitinib (Rux) treated samples and CM + Rux and CM + Rux + IKK-16 treated samples. Counts were normalized using the relative log expression normalization method and only genes with counts per million greater than one in two or more samples were kept. A gene was categorized as differentially expressed if the p-value was less than 0.05 after p-value adjustment and if the log2 fold change was greater than 0.5 or less than − 0.5. P-values were adjusted using the Benjamini & Hochberg method. GO term enrichment analysis and gene set enrichment analysis (GSEA) were done using the ClusterProfiler R package^35^. The hallmark gene set from the Molecular Signatures Database v 7.4 (https://www.gsea-msigdb.org/gsea/msigdb/index.jsp) was used in the GSEA analysis. Human gene orthologs of mouse genes were obtained from the Mouse Genome Informatics website (http://www.informatics.jax.org/downloads/reports/HMD_HumanPhenotype.rpt). Pathway analysis on significantly upregulated genes was done using DAVID Functional Annotation Tool and Microarray analysis (https://david.ncifcrf.gov/)^36,37^. Transcription factor analysis was performed using the LISA Cistrome Analysis tool (http://lisa.cistrome.org/)^38^. Volcano plots for differential gene expression were created using VolcaNoseR (https://huygens.science.uva.nl/VolcaNoseR/)^39^. Gene Venn Diagrams were created using Venny^40^.

### Statistical analysis

All experiments were performed at least 3 times. Statistical analyses for 3 or more conditions were performed using one-way ANOVA with Tukey’s multiple comparison test. Patterns of tumor growth were compared by using two-way ANOVA with Tukey’s multiple comparison test on each time point. Bar graphs represent means ± SEM. Kaplan Meier curves were compared by using log-rank tests (GraphPad prism v9). For analyses of differentially expressed genes from RNA-seq results, Student’s unpaired, two-tailed t test was used with Tukey’s multiple comparison test.

## Results

### Macrophages mediate resistance of the tumor cells to JAK inhibitor ruxolitinib

**Figure 1:**
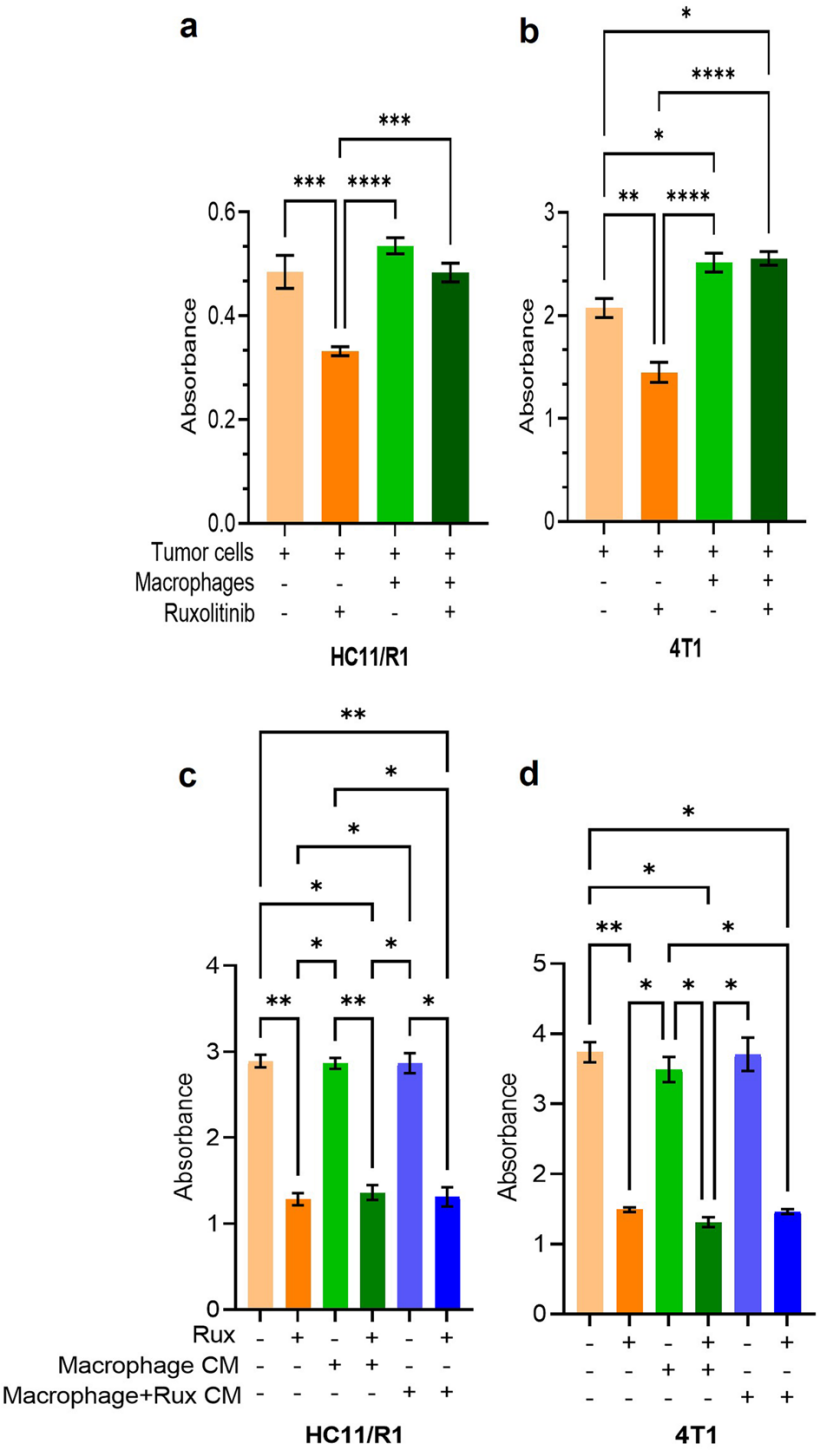
Macrophages mediate resistance of the tumor cells to JAK inhibitor ruxolitinib. Cell survival was measured by Crystal Violet Assay and absorbance was measured at 590 nm for HC11/R1 (**a**) and 4T1 (**b**) cells co-cultured with macrophages. HC11/R1 (**c**) and 4T1 cells (**d**) were treated with conditioned media from macrophages treated with ruxolitinib only, without tumor conditioned media. Tumor cell survival was measured using crystal violet assay *p < 0.05, **p < 0.01, ***p < 0.001, ****p < 0.0001, n = 3 biological replicates.

Numerous inhibitors of the JAK/STAT pathway are being investigated for breast cancer and importantly as targeted therapy for triple negative breast cancer. We have previously demonstrated that macrophages contribute to resistance to the JAK1/2 inhibitor ruxolitinib in human breast cancer cell lines^18^. To extend these findings to include additional models, we assessed the effect of macrophages on mouse mammary tumor cell lines treated with ruxolitinib. We used the mouse tumor cell line 4T1 and the previously characterized cell line HC11/R1, which contains a chemically inducible FGFR1 construct^22^. Dimerization and activation of the inducible FGFR1 can be achieved by treating HC11/R1 cells with the chemical homodimerizer B/B^22^. Treatment with B/B in vivo promotes migration, invasion, and tumor growth^22^. We have previously demonstrated that soluble factors from 4T1 and HC11/R1 cells activate the JAK/STAT pathway in macrophages^18,41^. We treated 4T1 cells and HC11/R1 cells with ruxolitinib in the presence of BMDMs plated in a transwell, also treated with ruxolitinib as shown in Supplementary Fig. S1a. We measured tumor cell viability using a crystal violet assay. While treatment of tumor cells with ruxolitinib reduced viability of both the 4T1 and HC11/R1 cells, we observed increased viability of both cell lines upon treatment with ruxolitinib in the presence of macrophages (Fig. 1a,b). We used increasing concentrations of ruxolitinib to assess survival and found that ruxolitinib was able to induce cell death in tumor cells at concentration as low as 0.5 μM (Supplementary Fig. S1c,d). This finding indicates that soluble factors released by macrophages promote resistance of the tumor cells to JAK inhibition. We also treated macrophages with ruxolitinib in absence of tumor CM and studied the efficacy of ruxolitinib on tumor cells in presence of the supernatant from macrophages (Supplementary Fig. S1b–d). Soluble factors from macrophages not treated with tumor CM were not able to reduce efficacy of ruxolitinib, indicating that tumor-derived factors are necessary to induce a tumor-promoting response in macrophages.

### Macrophages upregulate a subset of genes associated with tumor promotion in response to JAK inhibition

**Figure 2:**
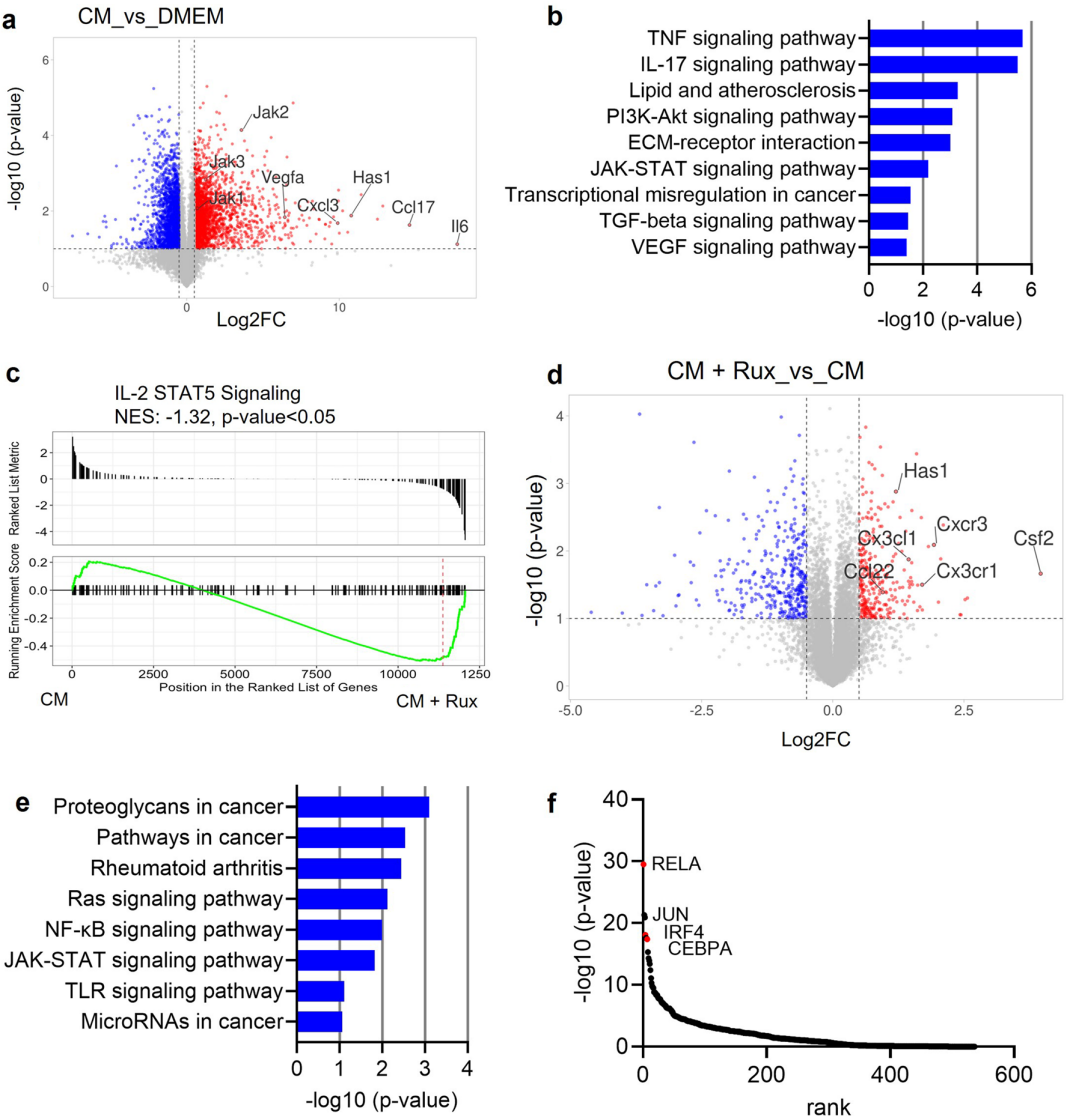
Macrophages upregulate a subset of genes associated with tumor promotion in response to JAK inhibition. (**a**) Volcano plot of differentially expressed gene in macrophages treated with tumor conditioned media (CM) from 4T1 cells, compared to DMEM. Genes with log_2_FC ≥ 0.5, p-value ≤ 0.05 were included in downstream analyses (**b**) Pathway analysis on upregulated genes from (**a**), using DAVID Pathway analysis tool. (**c**) Changes in expression of the IL-2 STAT5 signaling pathway in macrophages treated with tumor CM and vehicle vs CM and ruxolitinib (rux). (**d**) Volcano plot showing differentially expressed genes in macrophages treated with CM in presence of vehicle or ruxolitinib. Genes with log_2_FC ≥ 0.5 and p-value ≤ 0.05 were used for downstream analyses. (**e**) Pathway analysis on upregulated genes from (**d**), using DAVID pathway analysis tool (**f**) Transcription factor analyses on upregulated genes from (**d**) using the Lisa Cistrome analysis tool.

Further studies were performed to elucidate the gene expression profiles of macrophages in response to soluble factors from tumor cells and in presence of JAK inhibition. First, we treated macrophages, in the presence of conditioned media (CM) from 4T1 tumor cells, to study the effect of tumor-derived soluble factors on macrophages. Gene expression and pathway analysis showed that tumor CM activated a number of pathways in macrophages, such as the tumor necrosis factor pathway (TNF), PI3K-Akt pathway and the VEGF signaling pathway (Fig. 2a,b). Consistent with our previous results^18^, we also observed that CM treatment activated the JAK-STAT pathway in macrophages, making them susceptible to JAK inhibition. Next, to study changes induced by JAK inhibition specifically in tumor-associated macrophages, we treated macrophages with tumor-conditioned media in the presence of either vehicle or ruxolitinib. As expected, ruxolitinib treatment downregulated the expression of several genes in macrophages, including genes associated with the STAT5 signaling pathway (Fig. 2c,d). However, we also observed induction of a subset of genes as a result of JAK inhibition (Fig. 2d). Further analysis of these upregulated genes using the DAVID pathway analysis tool^36,37^ showed that ruxolitinib activated pathways in macrophages that are known to be associated with tumor-promoting function. For example, ruxolitinib treatment induced genes associate with the Ras/MAPK signaling pathway, which has been shown to promote tumorigenesis by driving the macrophages to a pro-tumor phenotype^42,43^ (Fig. 2e). Similarly, the NF-κB pathway was also significantly activated in response to JAK inhibition, which is known to drive cancer-related inflammation and tumor-promotion by myeloid cells in the tumor stroma^43–46^.Using the LISA Cistrome tool^38^, analysis of transcription factors potentially driving gene expression in ruxolitinib-treated macrophages demonstrated the RelA transcription factor to be the most common transcription factor associated with the upregulated genes (Fig. 2f). RelA (or p65) is a subunit of the NF-κB transcription factor complex that is phosphorylated and translocates to the nucleus to regulate transcription^19^. In concordance with these studies, analysis of data from our previous studies shows that RelA also regulates gene expression in human primary macrophages treated with MDA-MB-231-derived CM and ruxolitinib (Supplementary Fig. S2a,b)^18^. We thus hypothesized that ruxolitinib-mediated JAK inhibition activates the NF-κB transcription factor pathway in TAMs. We did not observe a similar induction of genes in macrophages treated with ruxolitinib in absence of tumor CM (data not shown), suggesting the requirement of tumor derived soluble factors in activating this transcriptional response in macrophages.

### NF-κB is activated in tumor-conditioned macrophages following treatment with JAK inhibitors

**Figure 3:**
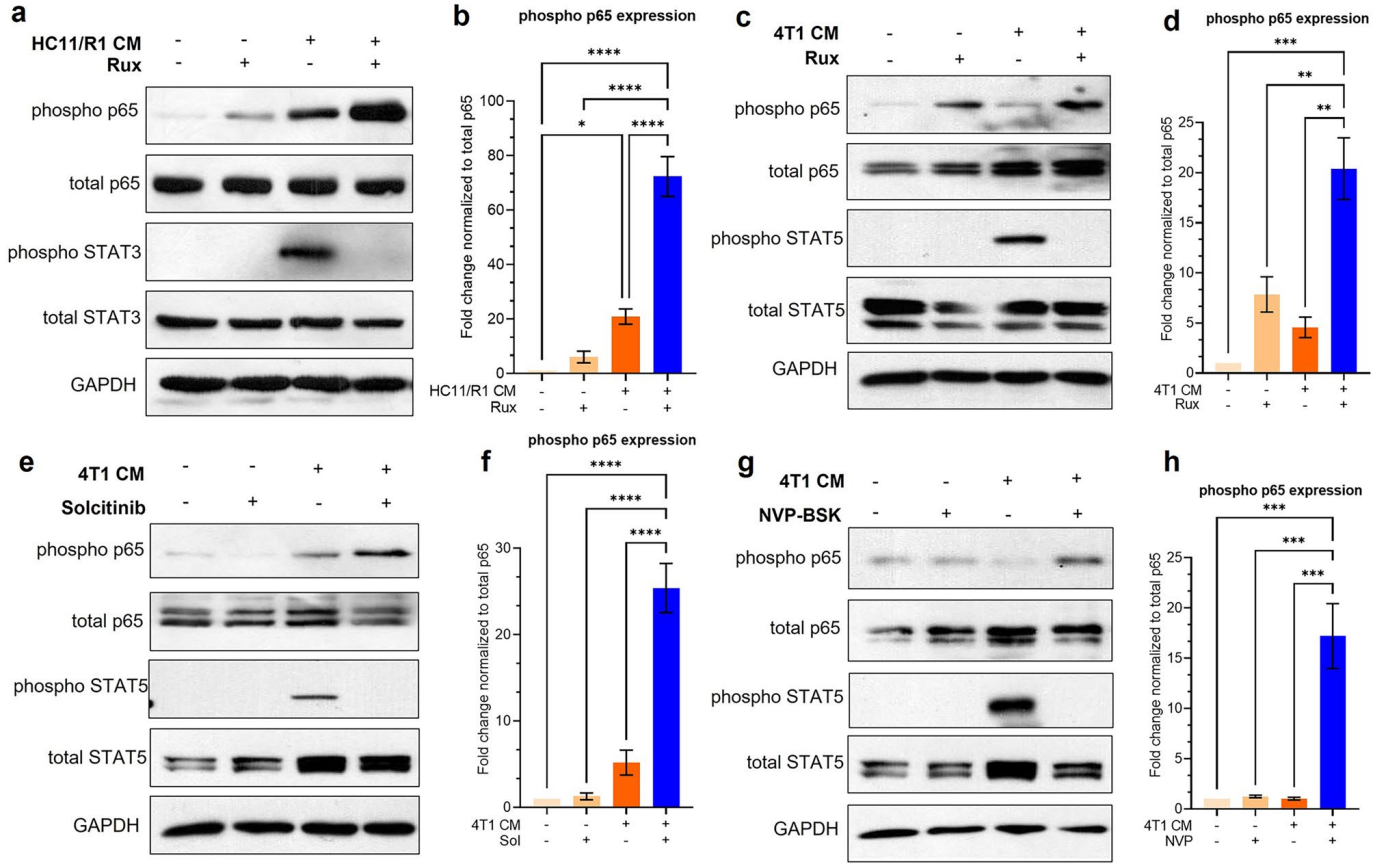
NF-κB is activated in tumor-conditioned macrophages treated with JAK inhibitors. (**a**–**d**) Immunoblot and quantification of protein lysates from BMDMs treated with CM from HC11/R1 cells (**a**,**b**) and 4T1 cells with ruxolitinib (**c**,**d**). (**e–h**) immunoblot of BMDMs treated with CM from 4T1 cells with solcitinib (**e**,**f**) and NVP-BSK805 (**g**,**h**). *p < 0.05, **p < 0.01, ***p < 0.001, ****p < 0.0001 Representative blots from n = 3 biological replicates.

**Figure 4:**
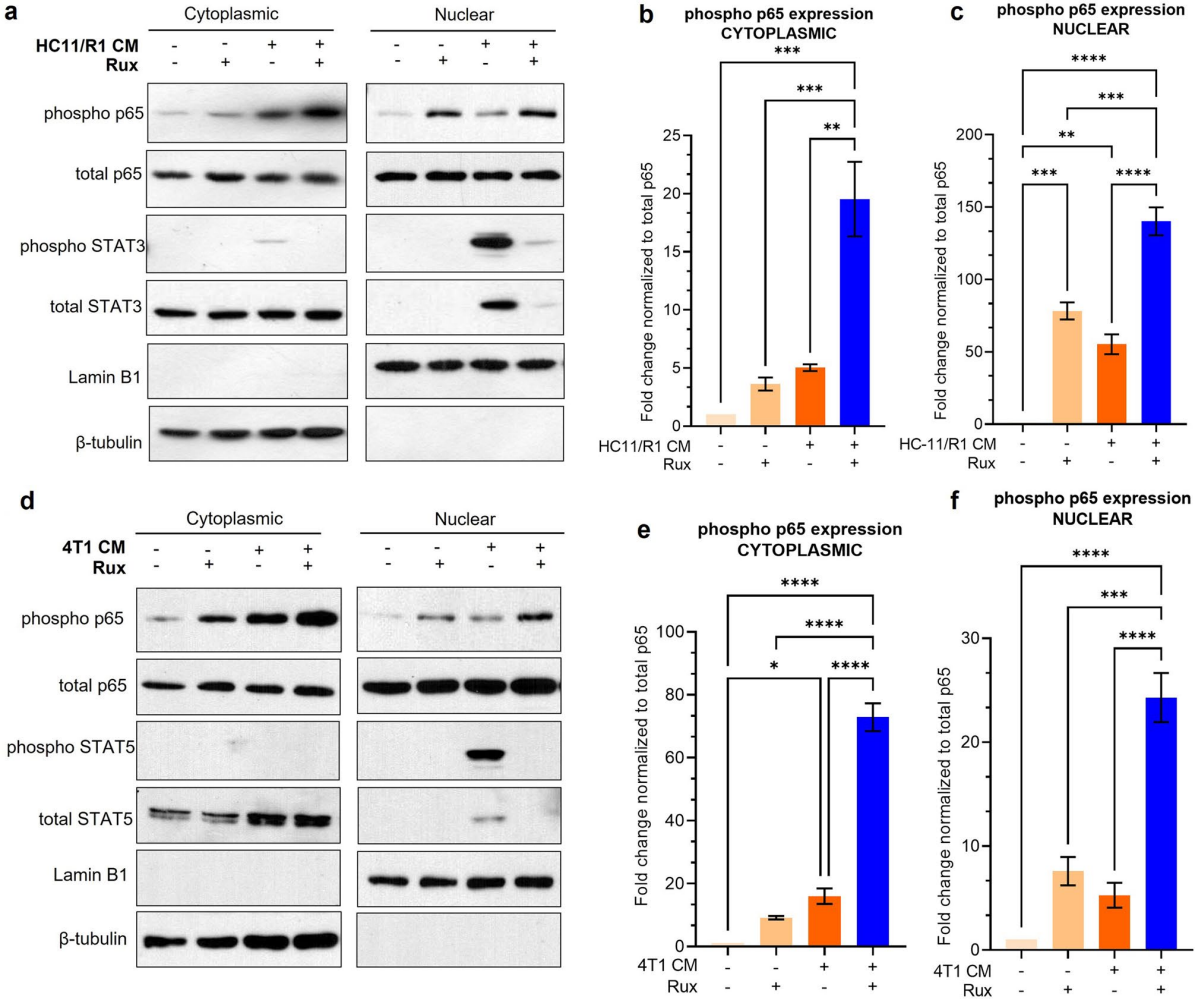
Ruxolitinib treatment increases nuclear localization of NF-κB in TAMs. BMDMs were treated with CM from HC11/R1 cells (**a**–**c**) and 4T1 cells (**d**–**f**) with ruxolitinib, and protein lysates from the cytoplasmic and nuclear fraction were immunoblotted for phospho-p65. *p < 0.05, **p < 0.01, ***p < 0.001, ****p < 0.0001 Representative blots from n = 3 biological replicates.

NF-κB is a critical transcription factor pathway predominantly activated downstream of the TNFα receptor^20^. The p65 and p50 subunits of NF-κB are phosphorylated by the upstream kinase Inhibitor of kappa kinase (IKK). The complex of p65 and p50 subunit then translocates to the nucleus to regulate transcription^20^. Our results suggest that NF-κB is activated in macrophages treated with ruxolitinib. To confirm this finding, we isolated BMDMs from WT Balb/c mice and treated them with CM from B/B-treated HC11/R1 (Fig. 3a,b) and 4T1 (Fig. 3c,d) tumor cell lines. We observed increased phosphorylation of the p65 subunit of NF-κB in macrophages treated with tumor cell CM in the presence of ruxolitinib. Both STAT3 and STAT5 are activated by 4T1 and HC11/R1 CM in BMDMs. However, HC11/R1 cells secrete significantly more IL-6 upon treatment with B/B than 4T1 cells, which robustly phosphorylates STAT3 in macrophages treated with CM^18,47^. Conversely, 4T1 cells secrete significant amounts of the cytokine GM-CSF, which we have previously shown to phosphorylate STAT5 in macrophages^18,41^. Therefore, we further investigated whether activation of STAT proteins in macrophages was downstream of JAK1/2. Immunoblot analysis of phospho-STAT3 in BMDMs treated with HC11/R1 CM, and for phospho-STAT5 in BMDMs treated with 4T1 CM demonstrated that phosphorylation of both the STAT proteins is inhibited by ruxolitinib (Fig. 3). Together, our data suggest increased phosphorylation of the p65 subunit of NF-κB following inhibition of JAK in tumor associated macrophages. To determine whether the activation is specific to JAK inhibition by ruxolitinib, we treated BMDMs with CM from 4T1 tumor cells in presence of Solcitinib (Fig. 3e,f) and NVP-BSK805 (Fig. 3g,h), inhibitors specific for JAK1 and JAK2 respectively^48,49^. We observed a similar increase in phosphorylation of p65 in BMDMs treated these inhibitors, suggesting that NF-κB activation is not specific to ruxolitinib-mediated JAK inhibition. Our results thus far suggest that NF-κB is activated in tumor associated macrophages treated with JAK inhibitors.

### Ruxolitinib treatment increases nuclear localization of NF-κB in TAMs

Upon phosphorylation, the p65 and p50 units of NF-κB translocate to the nucleus and bind to promoter regions of target genes to regulate transcription^20^. In order to determine if ruxolitinib treatment increases nuclear translocation of phosphorylated p65, we performed immunoblot analyses on cytoplasmic and nuclear fractions separately of BMDMs treated with HC11/R1 (Fig. 4a–c) and 4T1 (Fig. 4d–f) CM. We detected increased phosphorylated p65 in nuclear lysates of BMDMs treated with CM and ruxolitinib, suggesting increased nuclear translocation of activated NF-κB. Our results indicate that ruxolitinib leads to increased NF-κB activation and increases nuclear translocation in TAMs, thus also suggesting an increase in NF-κB transcriptional activity.

### Inhibition of NF-κB through the canonical pathway inhibits the induction of a subset of genes in TAMs treated with ruxolitinib

**Figure 5:**
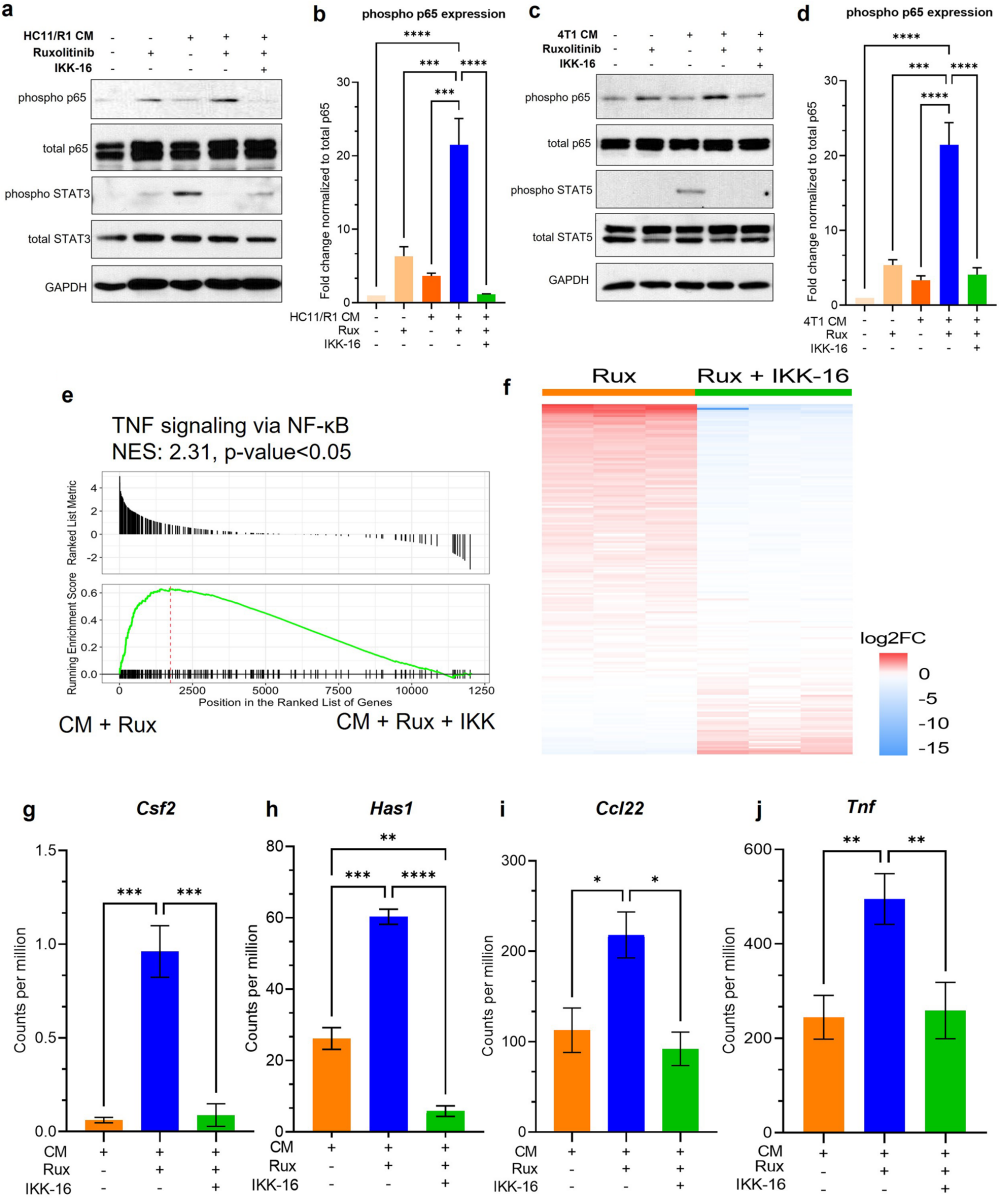
Inhibition of NF-κB through the canonical pathway inhibits the induction of genes in TAMs treated with ruxolitinib. Immunoblots of BMDM lysates treated with HC11/R1 CM (**a**,**b**) and 4T1 CM (**c**,**d**) with ruxolitinib and IKK-16. Representative blots from n = 3 biological replicates. (**e**) Changes in expression in TNF signaling via NF-κB pathway in 4T1 CM and ruxolitinib treated cells vs CM, ruxolitinib and IKK-16 treated cells. (**f**) Heatmap showing gene expression of upregulated genes from Fig. 1f in BMDMs treated with CM and ruxolitinib vs CM, ruxolitinib and IKK-16. (**g**–**i**) Gene expression of *Csf2*, *Has1*, *Ccl22 and Tnf* in macrophages treated with CM, ruxolitinib and IKK-16, plotted as counts per million from RNA-sequencing reads. *p < 0.05, **p < 0.01, ***p < 0.001, ****p < 0.0001.

The p65 subunit of NF-κB is phosphorylated by upstream kinases IKKα and IKKβ, by mediating degradation of the sequestering protein IκB as well as directly phosphorylating the S536 site on p65^19^. This activation mechanism is a part of the canonical NF-κB activation pathway^50^. While these results suggested increased phosphorylation of p65 at the S536 site, the upstream kinases responsible for the activation remained unclear. We treated BMDMs with IKK-16, an inhibitor of IKKα/β kinases, in addition to ruxolitinib and tumor cell-conditioned media. We found that IKK-16 inhibits the phosphorylation of p65 observed in BMDMs treated with HC11/R1 and 4T1 CM and ruxolitinib (Fig. 5a–d). This finding suggests that the p65 activation observed in ruxolitinib treated BMDMs is mediated through the canonical NF-κB pathway.

To study the effect of NF-κB inhibition on gene expression in macrophages, and to assess whether IKK affects the induction of genes observed in Fig. 2d, we treated BMDMs with IKK-16 in addition to CM and ruxolitinib and compared gene expression to macrophages treated without the NF-κB inhibitor. Macrophages treated with conditioned media with ruxolitinib showed a positive enrichment score for genes involved in the NF-κB pathway, in comparison to treatment with ruxolitinib and IKK-16 (Fig. 5e). This suggests inhibition of the NF-κB pathway by IKK-16 in macrophages. Comparing expression of the genes upregulated by ruxolitinib (Fig. 2d) to their expression when additionally treated by IKK-16 shows that NF-κB inhibition downregulates 50 percent of the genes induced by ruxolitinib alone (Fig. 5f). Several genes such as *Csf2, Has1*, *Ccl22* and *Tnf* induced by ruxolitinib and inhibited by IKK-16 (Fig. 5g–j) are associated with driving tumor-promoting functions in macrophages and increasing survival of tumor cells^51–57^. Taken together, our results suggest that the NF-κB pathway mediates upregulation of a gene expression profile associated with tumor progression in macrophages treated with JAK inhibitor and may be a mediator of macrophage derived resistance to JAK inhibition.

### NF-κB inhibition in combination with ruxolitinib improves therapeutic efficacy

**Figure 6:**
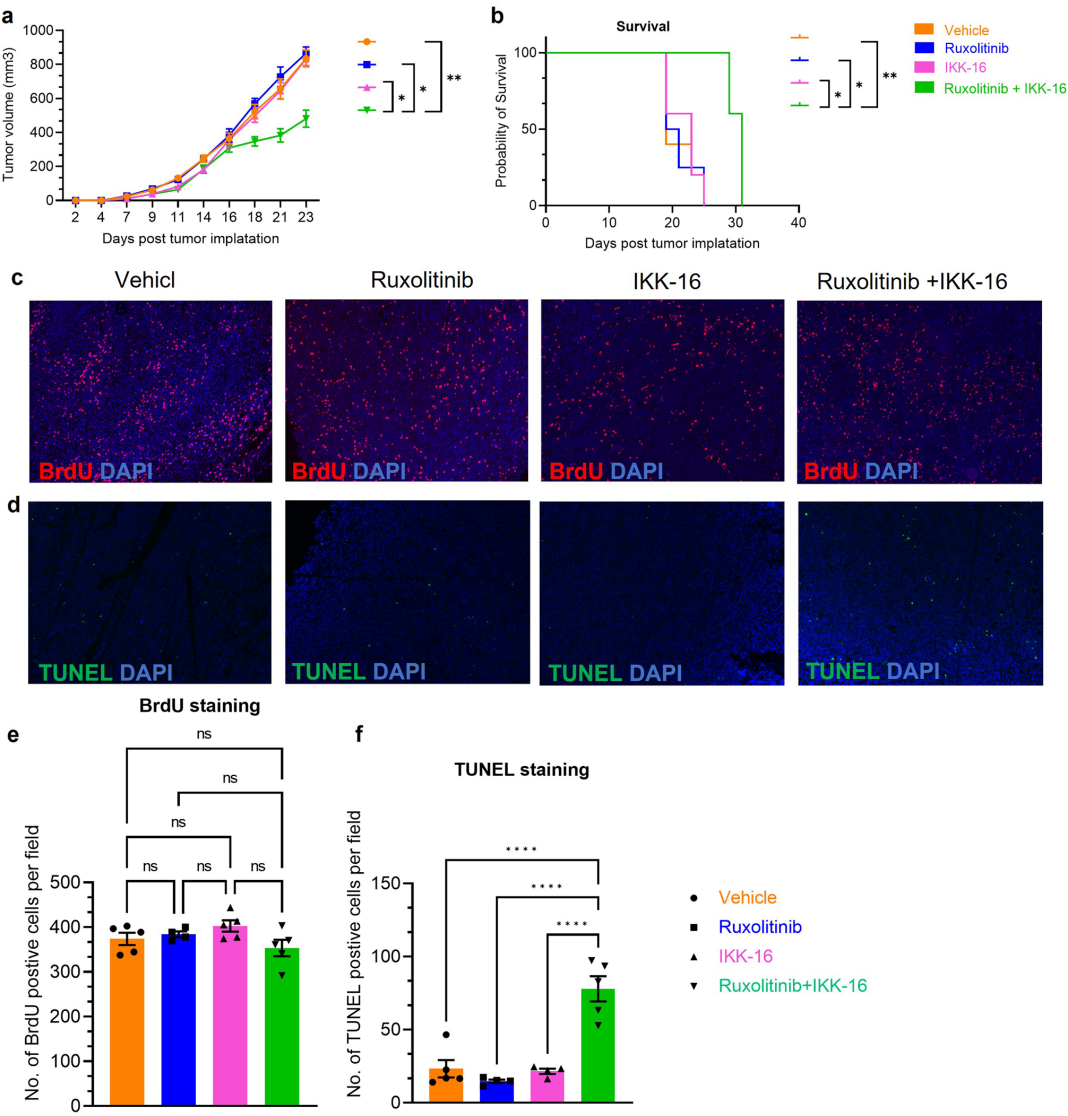
Inhibition of NF-κB in combination with JAK inhibition enhances responsiveness to ruxolitinib. (**a**) Tumor volume graph for each treatment group in 4T1 tumor bearing mice. Treatments were started when tumors reached 200mm^3^ and mice were euthanized at tumor size of 1000 mm^3^. (**b**) Kaplan–Meier curves of 4T1 tumor bearing mice for each treatment group. % survival on Y-axis indicates proportion of mice reaching tumor end point of 1000 mm^3^. (**c**–**f**) Representative immunofluorescent images and quantification of BrdU staining (**c**,**e**) (red) and TUNEL staining (**d**,**f**) (green) from tumor sections of 4T1 tumor bearing mice from each treatment group. 5 images were randomly captured from each tumor section and quantified using ImageJ. n = 4 for ruxolitinib treatment group, n = 5 mice for all other treatment groups. *p < 0.05, **p < 0.01, ***p < 0.001, ****p < 0.0001.

Our results thus far demonstrate that the NF-κB pathway mediates induction of a subset of genes in macrophages known to be associated with tumor progression. Based on these results, we hypothesized that NF-κB may be a possible mediator of resistance to ruxolitinib mediated by macrophages. We evaluated the effectiveness of a combination of NF-κB and JAK inhibition in vivo compared to single therapy with JAK inhibition. 4T1 tumor bearing mice were treated ruxolitinib and IKK-16 either alone or in combination. Tumor growth rates were not significantly different between vehicle control, ruxolitinib-only and IKK-16 only treatment groups (Fig. 6a). This is consistent with our previous results where we observed a lack of efficacy of ruxolitinib in vivo^18^. However, we observed significantly slower tumor growth rates in mice treated with ruxolitinib and IKK-16 together. Similarly, we observed improved survival in mice treated with the combination therapy compared to vehicle control and each inhibitor alone (Fig. 6b). While there was no significant difference in the extent of proliferation between the treatment groups, as measured by staining with the DNA ortholog BrdU (Fig. 6c,e), labeling of apoptotic cells using the TUNEL fluorometric system showed increased apoptosis in the tumors treated with the combination of ruxolitinib and IKK-16 (Fig. 6d,f). These data further corroborate the involvement of NF-κB in mediating resistance to JAK inhibition. These findings also suggests that IKK-16 helps combat the compensatory negative effects of macrophages that cause resistance to ruxolitinib, and inhibition of NF-κB in combination with ruxolitinib is beneficial over treating with ruxolitinib alone.

## Discussion

The TME and its impact on every aspect of tumor development is an immensely important consideration while developing anti-tumor therapies^58–61^. Our results from this and previous studies indicate that the JAK/STAT pathway is activated in tumor and stromal cells alike^18,47,62^. Targeting oncogenic pathways in tumor cells is accompanied by the unavoidable caveat of targeting the same pathways in cells of the TME. Our study shows that this off-cell-type targeting can lead to development of resistance to tumor-targeted therapy and targeting the TME in combination with the tumor cells may be beneficial.

Ruxolitinib, a JAK1/2 inhibitor is currently in clinical trials for ER+, HER2+ and triple negative breast cancer either as a single therapy or in combination with chemotherapy^9^. However, two clinical trials investigating ruxolitinib in triple negative breast cancer have been terminated due to lack of efficacy over placebo^63,64^. We have previously shown that soluble factors from macrophages promoted resistance to ruxolitinib in human breast cancer cell lines^18^. We have also shown that a combination therapy targeting the macrophage-derived soluble factors in addition to the tumor cells is beneficial over single treatment with JAK inhibitors in vivo^18^. We observed an improved response to ruxolitinib in our murine mammary tumor models when combined with macrophage depletion from the TME^18^. In the current study, our in vitro co-culture experiments indicate that the presence of macrophages increases survival in cells treated with ruxolitinib. We did not observe the same protective effect if the macrophages were treated with ruxolitinib in the absence of tumor conditioned media, suggesting that soluble factors from tumor cells were necessary for macrophages to mediate resistance to ruxolitinib. Gene expression studies of TAMs treated with ruxolitinib showed that mammary tumor cells activate the JAK/STAT pathway in TAMs via secreted soluble factors, and thereby activate several tumor promoting pathways such as the TNF signaling pathway, PI3-Akt signaling pathway, VEGF signaling pathway^65,66^. Inhibiting the JAK pathway in the TME also targets the TAMs, and our studies have shown that in macrophages this upregulates a subset of genes enriched in cancer associated pathways and known to be associated with tumor promotion^42,65^. Of note, we did not observe upregulation of genes in macrophages treated with ruxolitinib without tumor-conditioned media, further corroborating the requirement of tumor-derived soluble factors. In this study we aimed to elucidate the mechanism underlying the activation of a gene signature observed in macrophages following JAK inhibition.

The NF-κB transcription factor pathway emerged as the most upregulated pathway when we analyzed our gene set via the DAVID and LISA pathway analysis^36–38^. In our previous study, we observed a similar phenotype in human macrophages treated with tumor cell-derived CM, which was also regulated by NF-κB. While there were few individual genes common between the two studies, likely owing to species differences in tumor and macrophage models used, the transcriptional analyses identifying NF-κB as the regulating transcription factor were in concordance, further corroborating our hypotheses. In this study, we observed increased phosphorylation of p65 at S536 site within 15 min after treatment in BMDMs treated with tumor CM and ruxolitinib, which suggests an interaction upstream of transcriptional regulation in the nucleus. We also observed an increase in p65 phosphorylation when the macrophages were treated with ruxolitinib in absence of tumor CM, which did not translate to an increased gene expression profile similar to the one observed in presence of CM (data not shown). Other JAK inhibitors such as Solcitinib and NVP-BSK805 also induced phosphorylation of p65 in TAMs, suggesting that this mechanism is not specific to JAK inhibition by ruxolitinib. In several cancer models, activation of NF-κB in stromal cells has been shown to be required for onset of tumorigenesis^43–46^. Release of cytokines such as TNF-α and IL-6 following NF-κB activation activates pro-survival signals in tumor cells and promotes growth and progression^67^. Inhibition of these inflammatory cytokines in a mouse model of hepatocellular carcinoma lead to decrease tumor load and delay in tumor onset^68^. Whereas genetic deletion and inhibition of NF-κB led to an anti-tumor effect in these models, defective NF-κB activity was also reported in tumor associated macrophages from a late stage murine sarcoma model, and restoring NF-κB activity was associated with tumor regression in a mouse mammary carcinoma model^69–71^. The role of NF-κB in the functions of macrophages in the tumor microenvironment thus seems to be dependent on tumor type, stage, and progression. Our gene expression studies in tumor-associated macrophages demonstrate that ruxolitinib treatment in presence of CM activated genes associated with the NF-κB pathway, several of which were associated with tumor-promoting function. Ruxolitinib upregulated expression of *Csf1*, widely associated with tumor promoting phenotype of macrophages^72–74^. Numbers of circulating tumor cells and metastases were shown to be reduced by genetic ablation of *Csf1*, a major growth factor required for macrophage differentiation^75^. Expression of several other genes upregulated by ruxolitinib such as *Csf2, Cx3cl1, Cx3cr1, Has1, and Ccl22* in myeloid cell populations is known to be associated with tumor progression, myeloid cell accumulation, and tumor progression^51–56,76,77^. This unforeseen effect needs to be considered during development and investigation of other JAK inhibitors as potential therapeutic options. CSF2 has been shown to mediate resistance to Csf1r inhibition, and to induce accumulation of tumor-promoting myeloid cells^51,52^. In mouse models of skin carcinogenesis, the CX3CL1-CX3CR1 axis was found to be important for promoting recruitment of tumor associated macrophages and promoted tumor progression^76^. HAS1 is an enzyme involved in expression of hyaluronan, an important component of the extracellular matrix, and a hyaluronan-rich tumor microenvironment is associated with enhanced malignancy and poor prognosis in cancer patients^56,77^. CCL22 is an important chemokine involved in recruitment of regulatory T cells, and blocking the CCL22-CCR4 axis inhibited tumor progression in in vitro and in vivo models. The effect of macrophages on mediating resistance to ruxolitinib may be due to mechanistic co-operation between these and several other factors, and further studies are needed to determine the role of specific soluble and intracellular factors.

IKK-16, an inhibitor of the kinase upstream of p65, IKKα/β, inhibited the phosphorylation of p65 by ruxolitinib, suggesting that the observed induction may be regulated by the canonical NF-κB pathway. Gene expression studies using RNA-sequencing in cultured BMDMs treated with CM and inhibitors, either alone or in combination, demonstrate that ruxolitinib treatment in presence of CM induced expression of 189 genes, 90 of which were significantly inhibited by IKK-16. This finding corroborates our hypothesis that the NF-κB pathway is one of the major regulators of the gene expression profile induced in TAMs by JAK inhibition. We observed an improved response to ruxolitinib when combined with IKK-16 in vivo, providing further evidence of the NF-κB pathway mediating resistance to ruxolitinib. Our studies demonstrated an increase in *Tnf* expression with ruxolitinib, which was inhibited by IKK-16. Ligands from the TNF superfamily are known to promote tumor cell proliferation and progression and induce tumor-promoting inflammation^57^. The ability of the NF-κB inhibitor to decrease TNF expression by macrophages and also inhibit downstream signaling in tumor cells may be a potential mechanism by which NF-κB inhibition improves efficacy of ruxolitinib in combination. Treating with IKK-*16 *in vivo is likely to target the entire TME in addition to macrophages, thereby inhibiting NF-κB in tumor cells as well. In a study with glioblastoma cell lines U251-MG and U87-MG, increased phosphorylation of p65 was observed after treatment with STAT3 inhibitor JSI-124, which is suggestive of a similar mechanism being activated in breast tumor cells^78^. Further studies are therefore needed to determine whether JAK inhibition induces a similar NF-κB-driven genetic profile in tumor cells, further contributing to resistance.

Several studies have described interactions between NF-κB and members of the JAK/STAT pathway at various interfaces in the cell. Members of the NF-κB pathway such as RelA have been shown to physically interact with STAT proteins such as STAT3, resulting in either co-operative transcriptional synergy or repression of transcription^79–83^. STAT5 inhibition can inhibit expression of NF-κB by downregulating expression of the NF-κB activator BCL10^84^. STAT5b has also been shown to inhibit NF-κB mediated signaling via protein interactions and limiting access to coactivators^85^. Genetic ablation of STAT5 in leukemia cells showed that STAT5 deregulated expression of NF-κB target genes by binding to promoter regions of overlapping target genes^86^. The co-operative as well as co-repressive nature of STAT and NF-κB pathways seems to be cellular context and stimulus dependent. We also observed increased activation of the TNF-NF-κB pathway in our myeloid specific STAT5 deletion model^41^. Similar to these observations, in TAMs with decreased STAT mediated transcription, NF-κB may upregulate expression of a subset of genes by binding to promoter regions of target genes in the nucleus. In our study, we observed increased phosphorylation of p65 within 15 min of treatment by tumor conditioned media and ruxolitinib, which begets the possibility that in our model, this interaction may be upstream of the nuclear function of these transcription factors.

The results from these studies support the concept that macrophages are key drivers of response to tumor-targeted therapy. Small molecule inhibitors are often developed and investigated with majority of their attention on the tumor cells. The signaling pathways are present and activated in most cells of the TME, and thus the effect of tumor-targeted inhibitors on the cells of the TME complicates the response to the inhibitors. Several other protein kinase inhibitors such as imatinib, temsirolimus, sorafenib and sunitinib have been shown to affect tumor-immune response by inhibiting critical pathways in T cells^87^. Special attention thus needs to be given to such counter-productive effects of inhibitors. Several inhibitors and antibodies targeting components of the NF-κB pathway are being investigated in pre-clinical and clinical models, making it an ideal target for exploring combination therapies^88,89^. Our studies with the JAK inhibitor ruxolitinib highlight an important mechanism employed by TAMs to create an immune suppressive environment around the tumor and mediate resistance to ruxolitinib. Development of novel inhibitor combinations targeting the TME in addition to the tumor cells may be beneficial for patients who do not respond to single therapy.

### Supplementary Information

Supplementary Figures.

## Data Availability

The datasets used and analyzed for bulk RNA sequencing experiments in the study are available in the NCBI GEO database under the accession number GSE218138.

## Abbreviations

JAK/STAT: Janus kinase/signal transducer and activator of transcription
NF-κB: Nuclear factor kappa-B
TME: Tumor microenvironment
TAM: Tumor-associated macrophage
CM: Conditioned media
BMDM: Bone marrow derived macrophages
GO: Gene ontology
GSEA: Gene set enrichment analysis
