# Author Correction: Targeting the NF-κB pathway enhances responsiveness of mammary tumors to JAK inhibitors

**DOI:** 10.1038/s41598-023-43916-y

**Published:** 2023-10-04

**Authors:** Aditi S. Bapat, Christine H. O’Connor, Kathryn L. Schwertfeger

**Affiliations:** 1 Molecular Pharmacology and Therapeutics Graduate Program, University of Minnesota, 2231 6th St SE, Minneapolis, MN 55455 USA; 2 University of Minnesota Supercomputing Institute, University of Minnesota, Minneapolis, MN USA; 3 Department of Laboratory Medicine and Pathology, University of Minnesota, Minneapolis, MN USA; 4 Masonic Cancer Center, University of Minnesota, Minneapolis, MN USA; 5 Center for Immunology, University of Minnesota, Minneapolis, MN USA

Correction to: *Scientific Reports* 10.1038/s41598-023-32321-0, published online 01 April 2023

The original version of this Article contained an error in Figure 3, panel g, where the phospho STAT5 band was incorrectly located in the second lane. The original Figure 3 and accompanying legend appear below.

**Figure 3:**
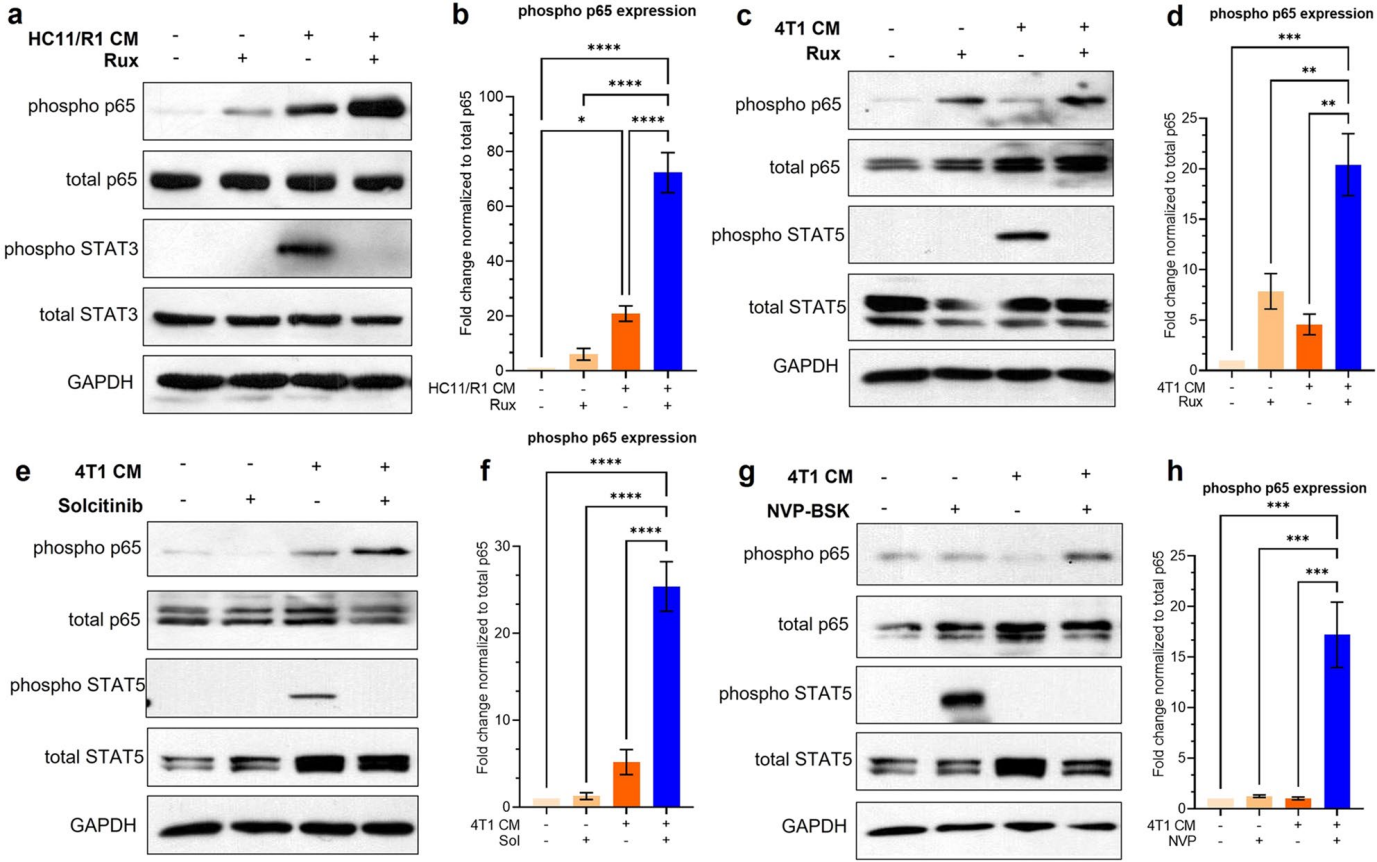
NF-κB is activated in tumor-conditioned macrophages treated with JAK inhibitors. (**a**–**d**) Immunoblot and quantification of protein lysates from BMDMs treated with CM from HC11/R1 cells (**a**,**b**) and 4T1 cells with ruxolitinib (**c**,**d**). (**e–h**) immunoblot of BMDMs treated with CM from 4T1 cells with solcitinib (**e**,**f**) and NVP-BSK805 (**g**,**h**). *p < 0.05; **p < 0.01; ***p < 0.001, ****p < 0.0001 Representative blots from n = 3 biological replicates.

The original Article has been corrected.

