# Supplementary material for: Targeting the NF-κB pathway enhances responsiveness of mammary tumors to JAK inhibitors
Source: Sci Rep. 2023 Apr 1;13:5349. doi: 10.1038/s41598-023-32321-0 (PMC10067805; doi:10.1038/s41598-023-32321-0)

S1

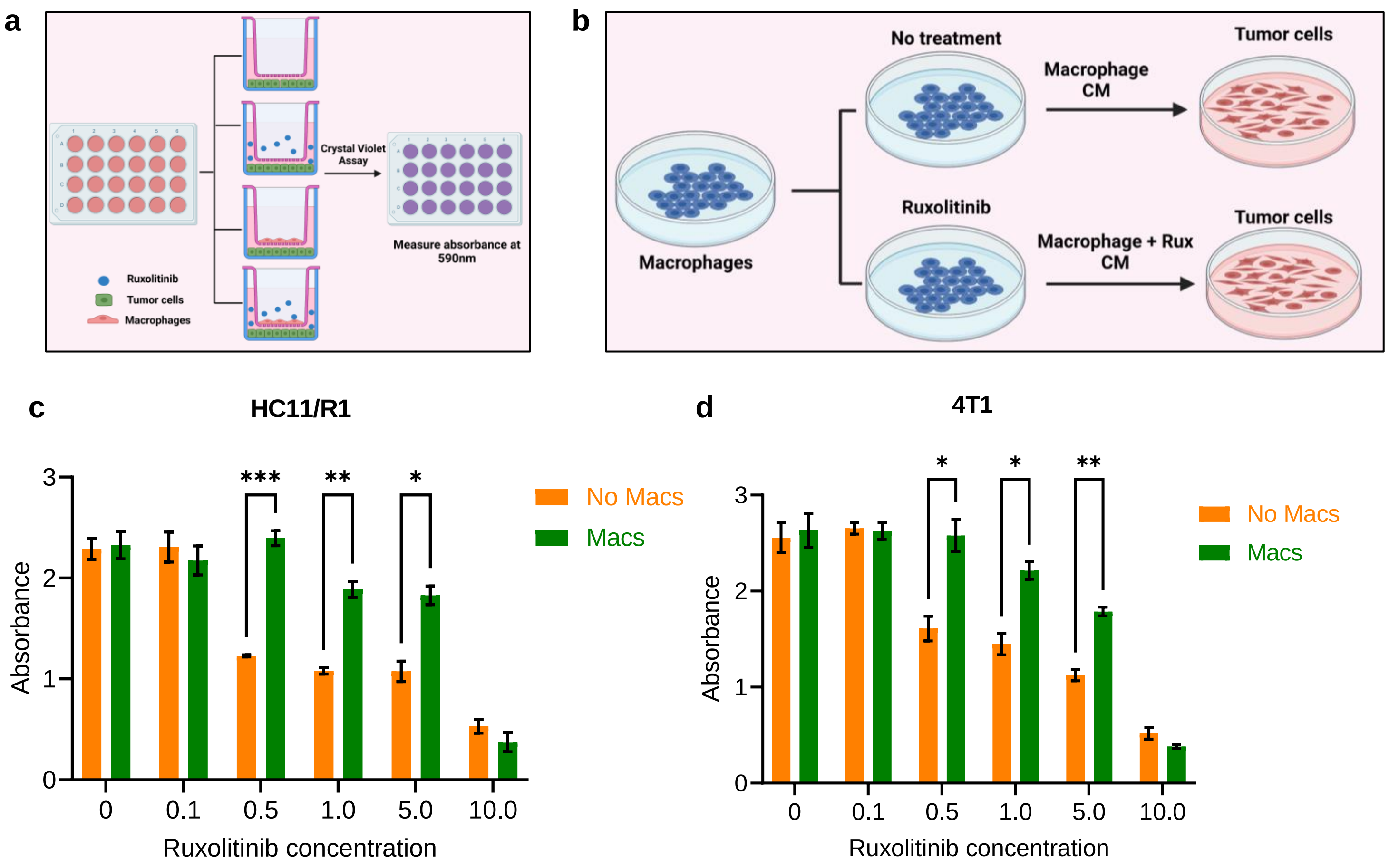

Figure S1: (a) Schematic of macrophages co-cultured with HC11/R1 and 4T1 tumor cells treated with ruxolitinib for 24 hours. Created using Biorender.com. **(b)** Schematic for tumor cells treated with conditioned media from ruxolitinib-treated macrophages for 24 hours. Created using Biorender.com. Cell survival measured by crystal violet assay for HC11/R1 **(a)** and 4T1 **(b)** cells co-cultured with macrophages and treated with different concentrations of ruxolitinib. **(c)** *p<0.05, **p<0.01, ***p<0.001, ****p<0.0001, n=3 biological replicates

S2

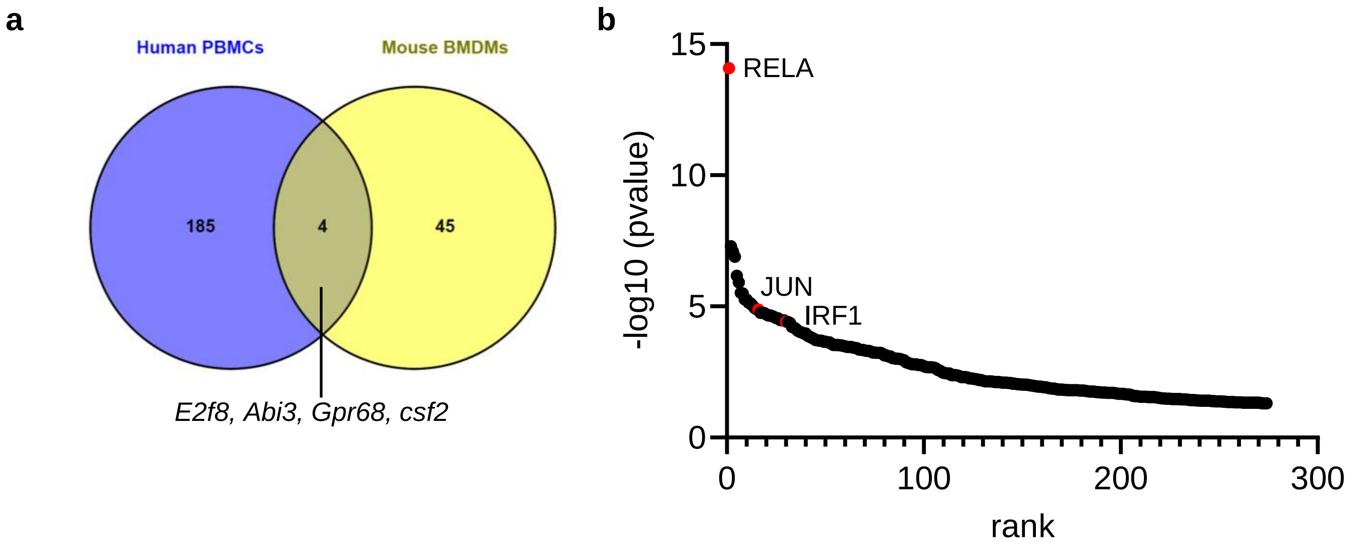

Figure S2: (a) Venn Diagram showing common genes upregulated in human PBMCs (blue) and mouse BMDMs (yellow) treated with tumor conditioned media and ruxolitinib **(b)** Transcription factor analyses on upregulated genes from human PBMCs in (a)**,** full gene list available in Irey, Lassiter et al, 2019 [18].

Representative uncropped blots for **Figure 3a**

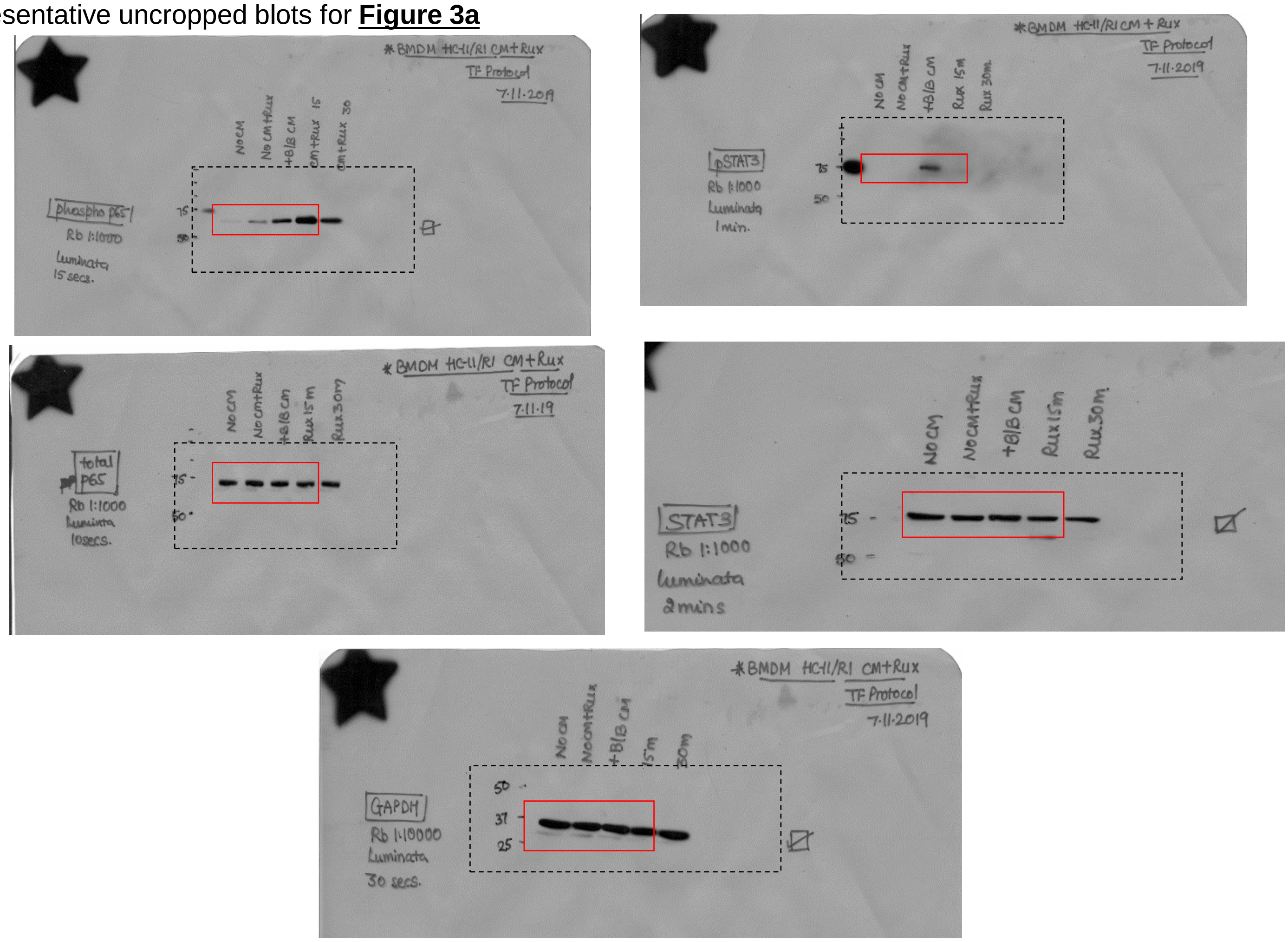

Representative uncropped blots for **Figure 3c**

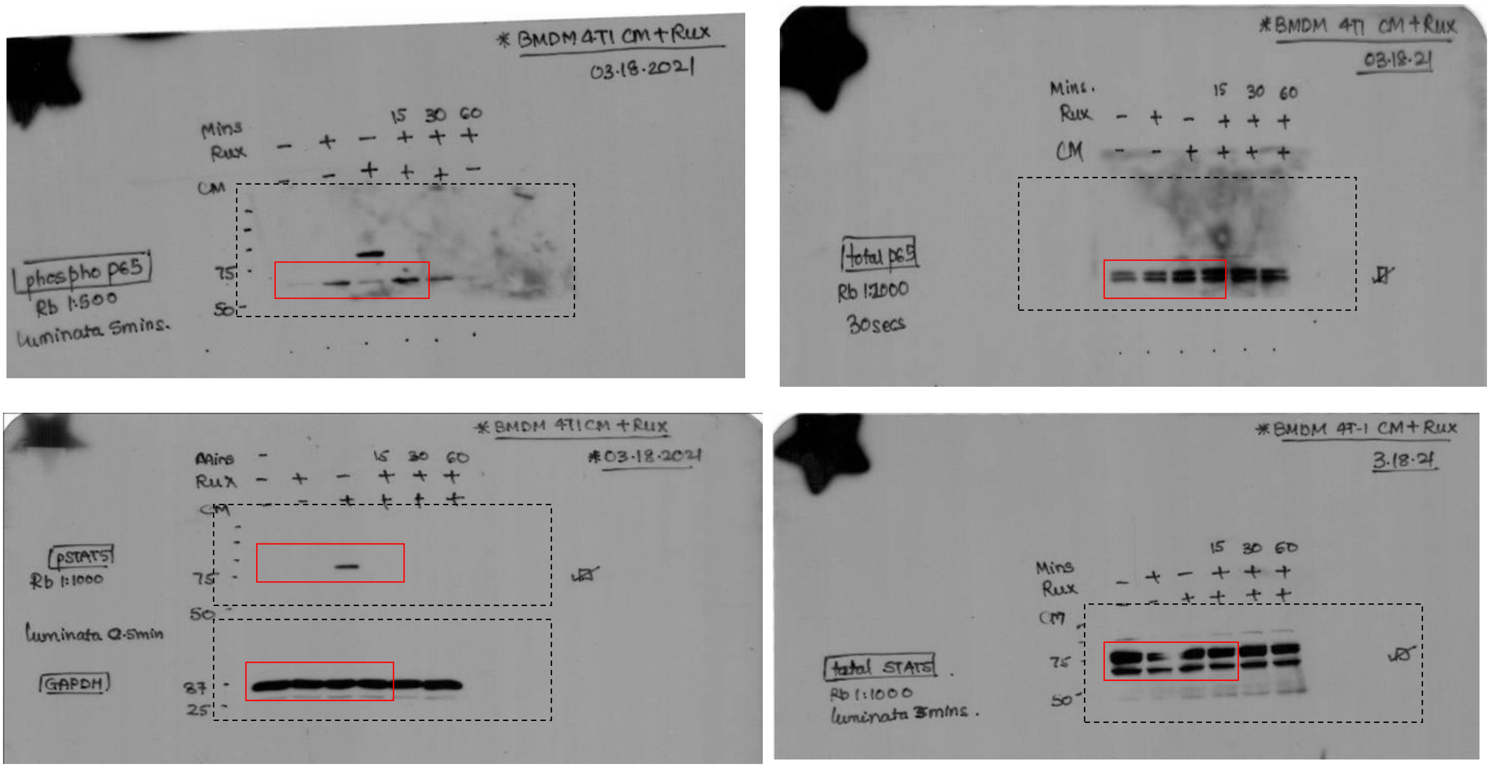

Representative uncropped blots for **Figure 3e and 3g**

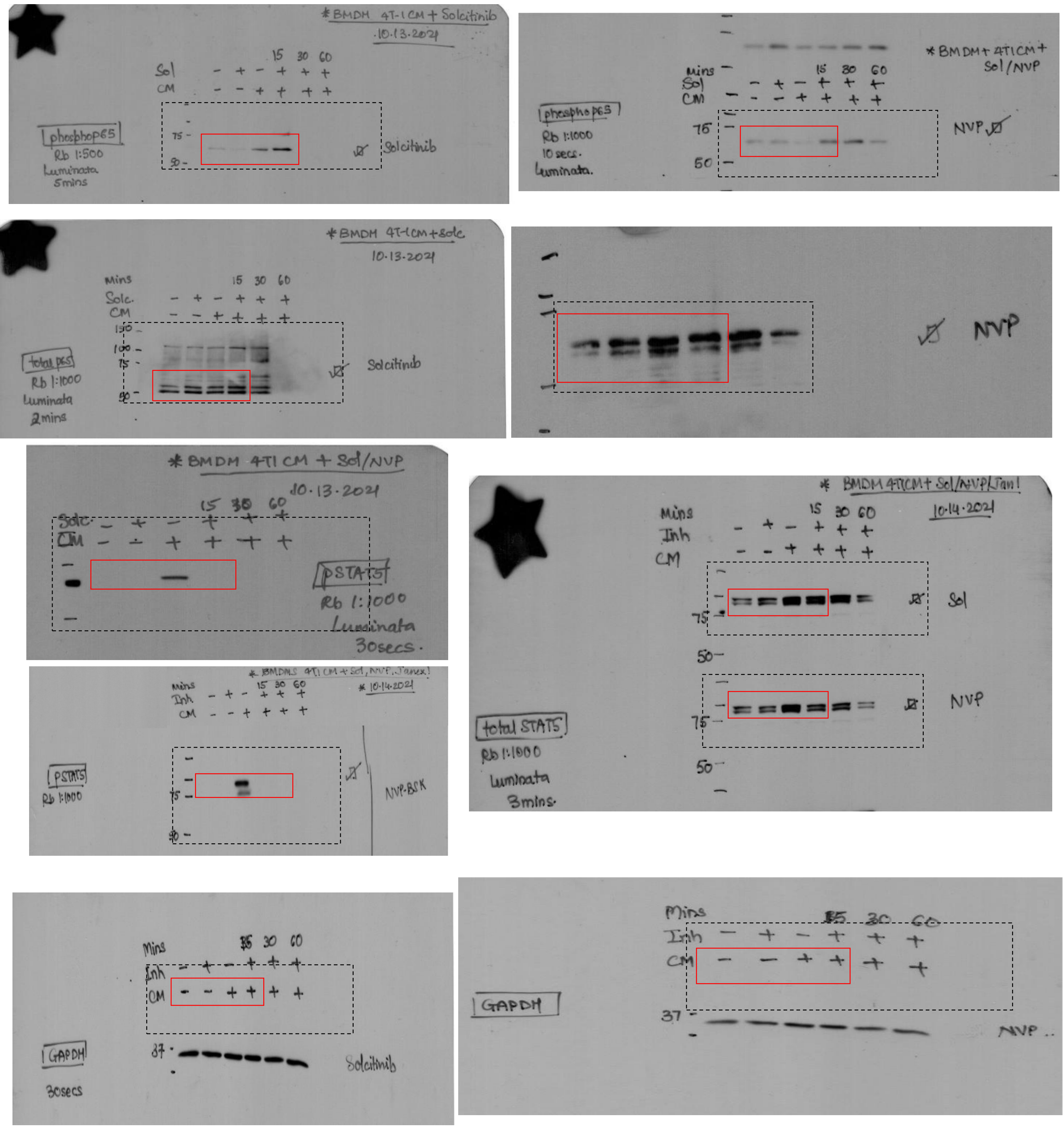

Representative uncropped blots for **Figure 4a**

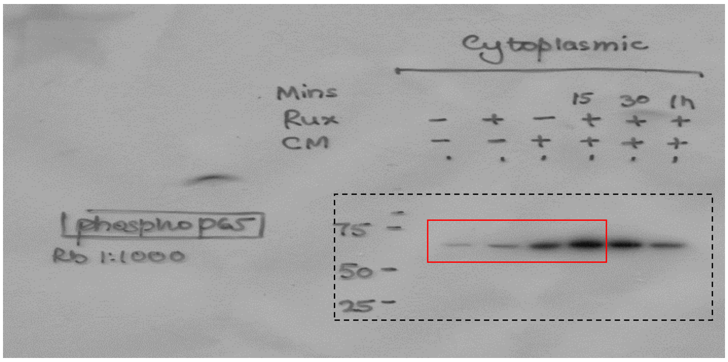

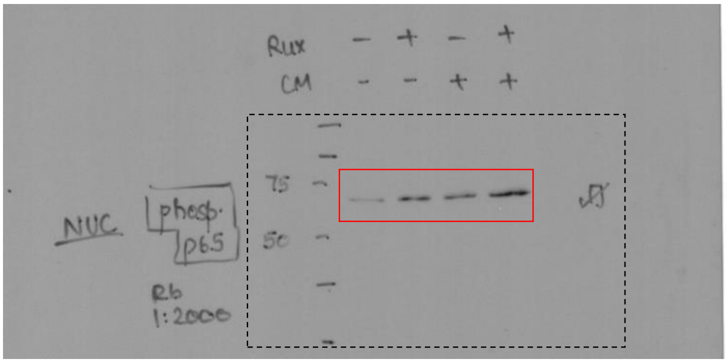

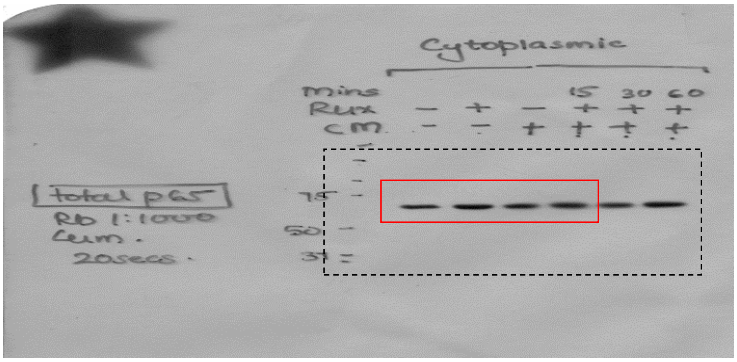

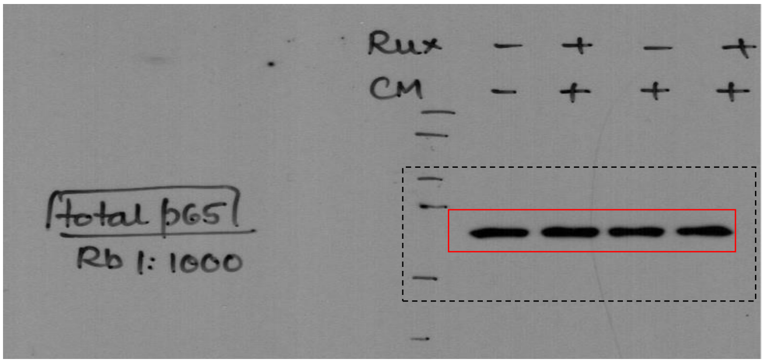

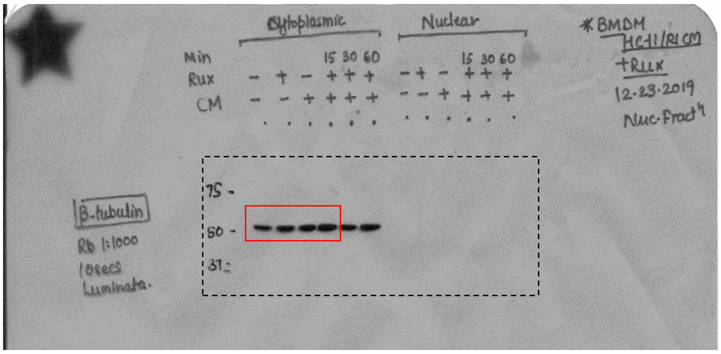

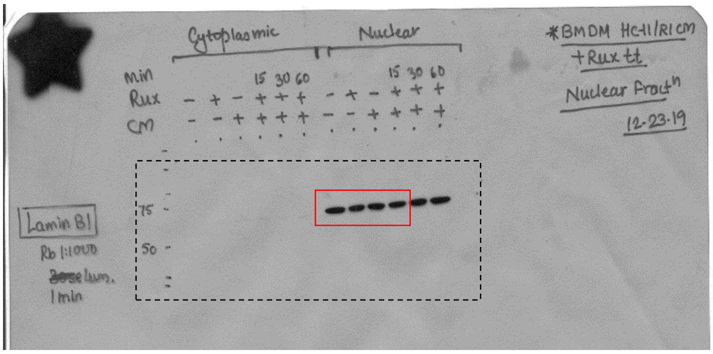

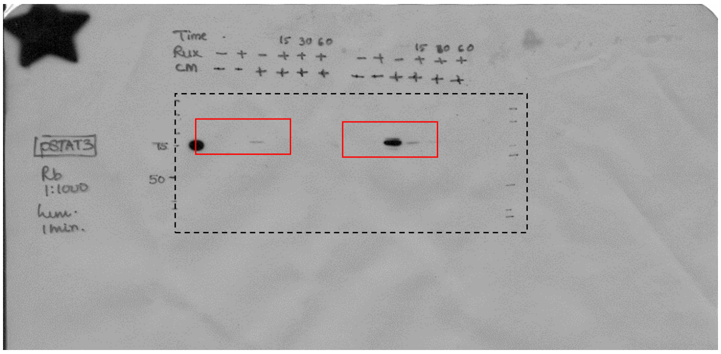

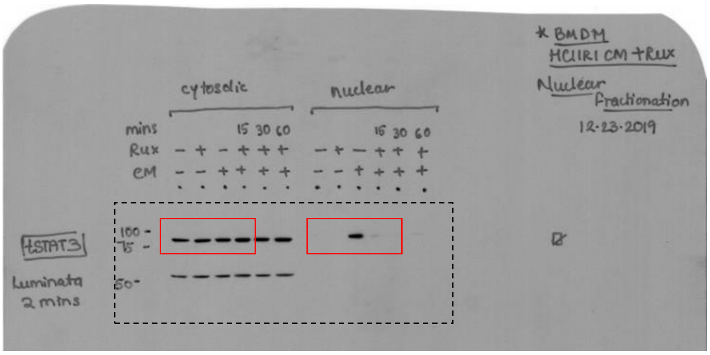

Representative uncropped blots for **Figure 4d**

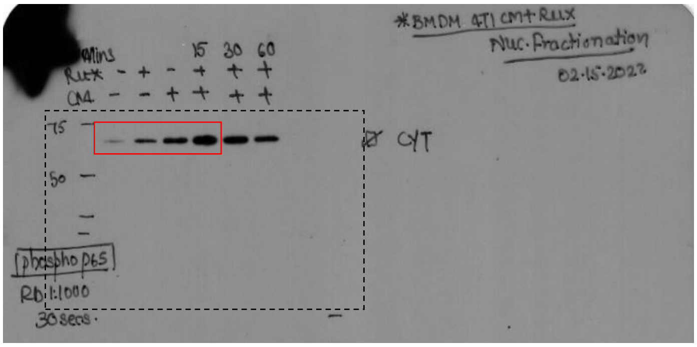

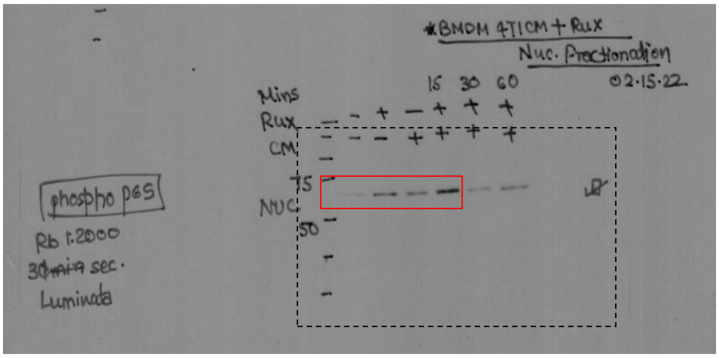

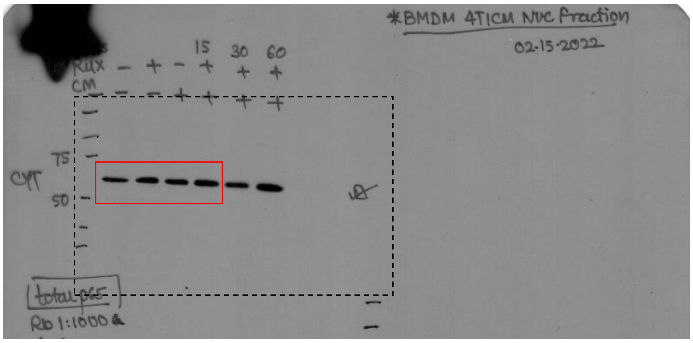

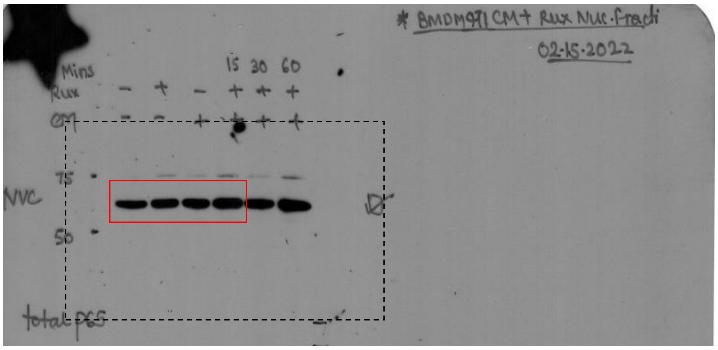

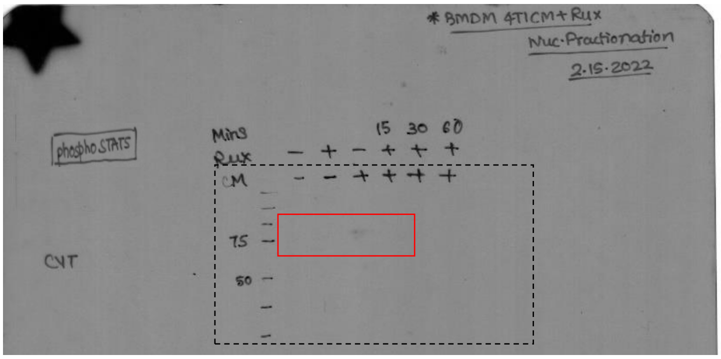

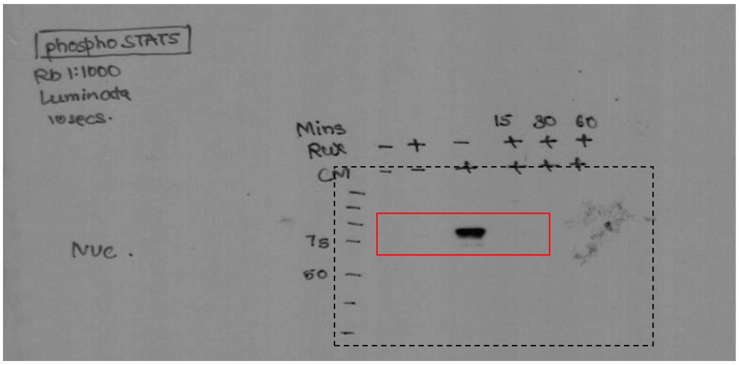

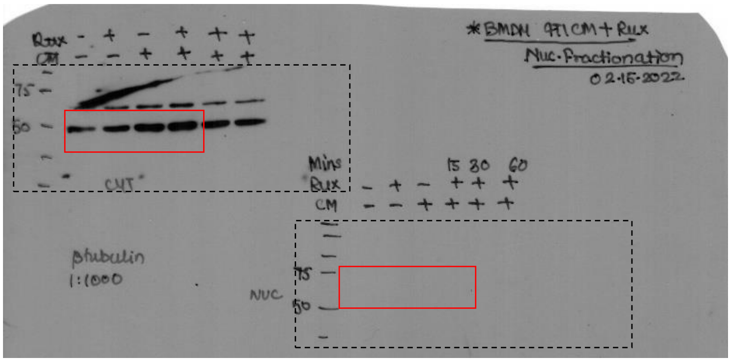

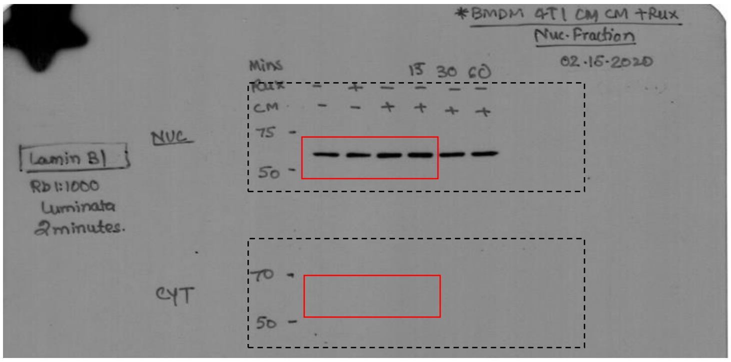

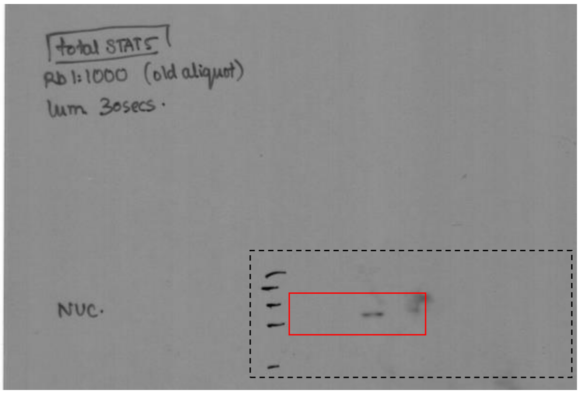

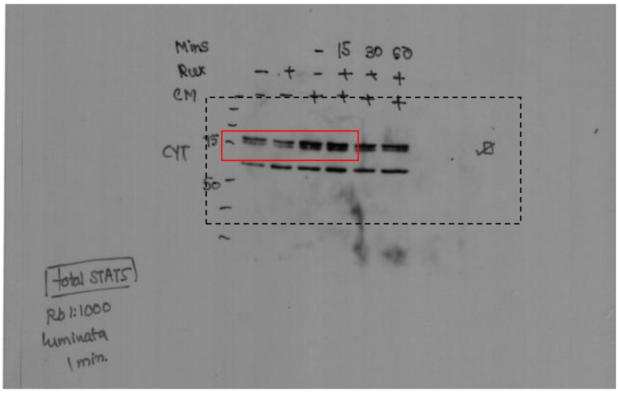

Representative uncropped blots for **Figure 5a**

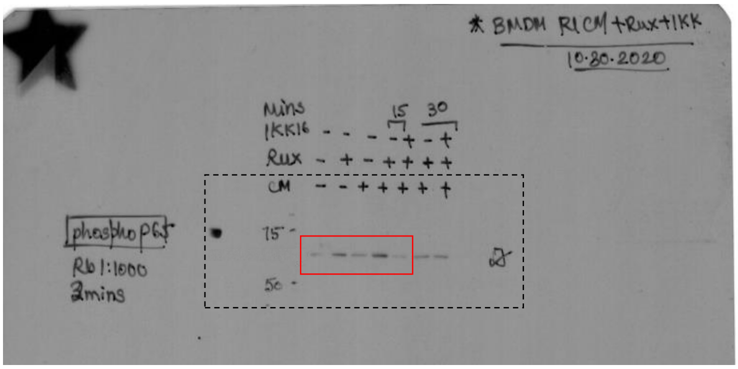

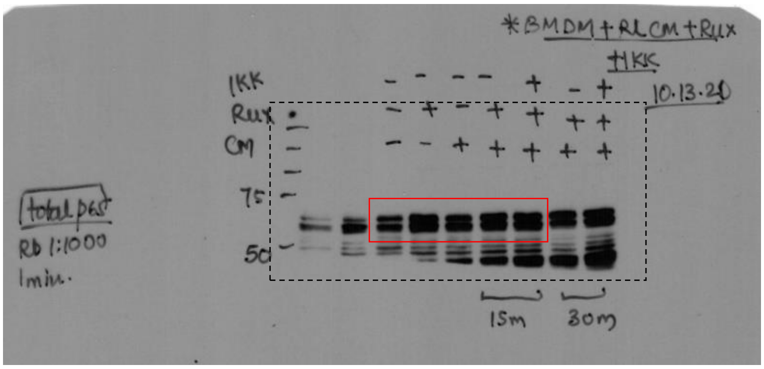

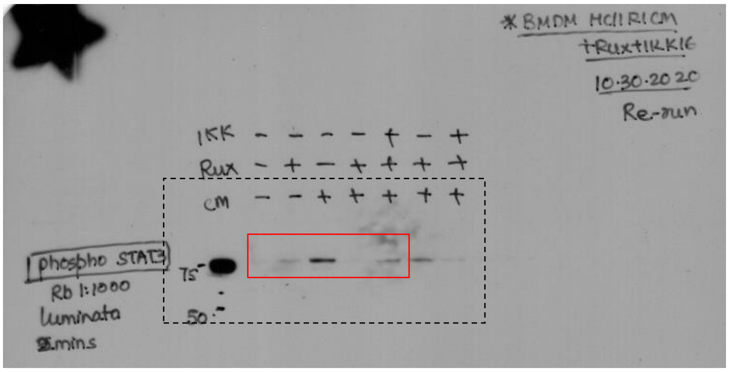

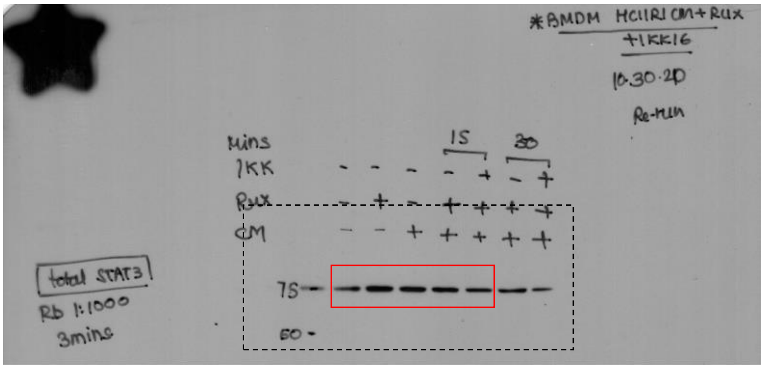

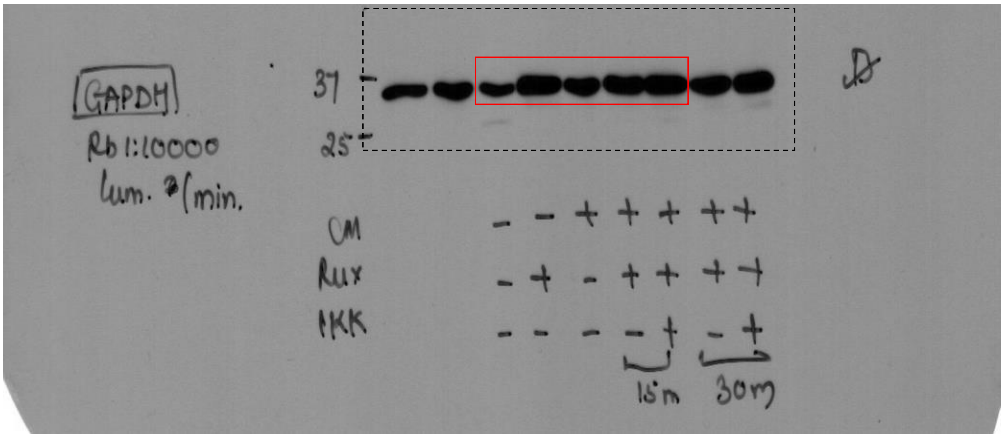

Representative uncropped blots for **5c**

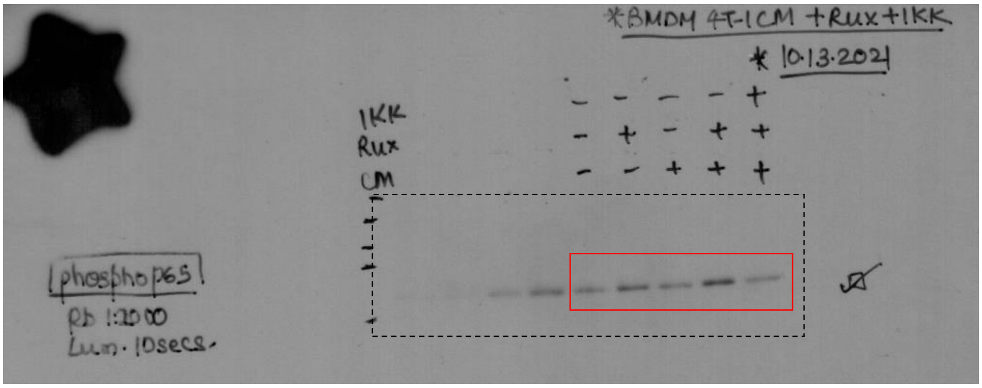

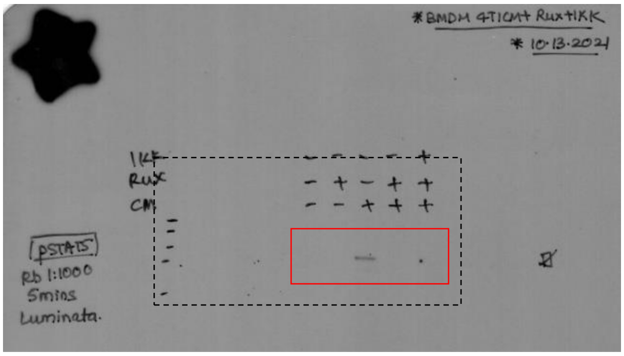

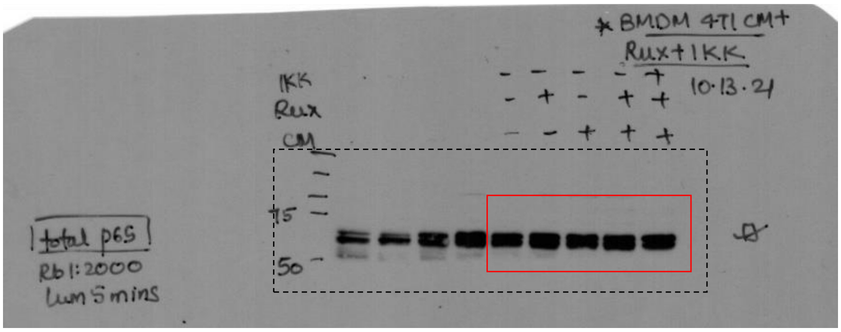

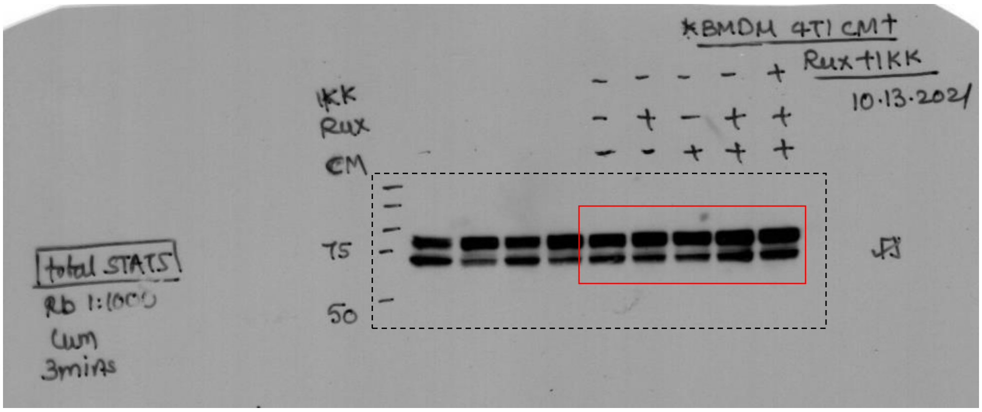

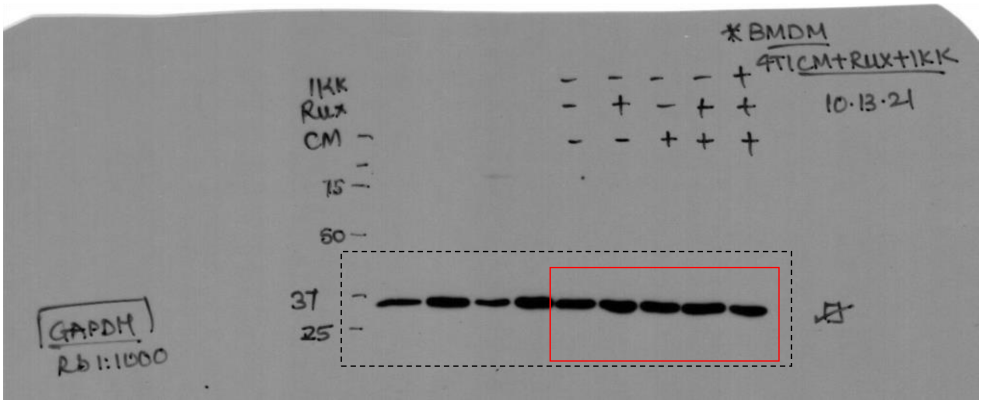

Supplement: Supplementary file 1 — Supplementary Figures. [file 41598_2023_32321_MOESM1_ESM.pdf]
